# GRP75-driven, cell-cycle-dependent macropinocytosis of Tat/pDNA-Ca^2+^ nanoparticles underlies distinct gene therapy effect in ovarian cancer

**DOI:** 10.1186/s12951-022-01530-6

**Published:** 2022-07-20

**Authors:** Linjia Su, Zhe Sun, Fangzheng Qi, Huishan Su, Luomeng Qian, Jing Li, Liang Zuo, Jinhai Huang, Zhilin Yu, Jinping Li, Zhinan Chen, Sihe Zhang

**Affiliations:** 1 Department of Cell Biology, School of Medicine, Nankai University, Nankai District, 94 Weijin Road, Tianjin, 300071 People’s Republic of China; 2 School of Life Sciences, Tianjin University, Weijin Road 92, Tianjin, 300072 China; 3 State Key Laboratory of Medicinal Chemical Biology, College of Chemistry, Nankai University, Weijin Road 94, Tianjin, 300071 China; 4 Department of Medical Biochemistry and Microbiology, Uppsala University, 75123 Uppsala, Sweden; 5 National Translational Science Center for Molecular Medicine, Department of Cell Biology, State Key Laboratory of Cancer Biology, Fourth Military Medical University, Xi’an, 710032 China

**Keywords:** Macropinocytosis, Cell-cycle, Glucose-regulated protein, Tat/pDNA-Ca^2+^ nanoparticle, Suicide gene therapy, Ovarian cancer

## Abstract

Practice of tumor-targeted suicide gene therapy is hampered by unsafe and low efficient delivery of plasmid DNA (pDNA). Using HIV-Tat-derived peptide (Tat) to non-covalently form Tat/pDNA complexes advances the delivery performance. However, this innovative approach is still limited by intracellular delivery efficiency and cell-cycle status. In this study, Tat/pDNA complexes were further condensed into smaller, nontoxic nanoparticles by Ca^2+^ addition. Formulated Tat/pDNA-Ca^2+^ nanoparticles mainly use macropinocytosis for intercellular delivery, and their macropinocytic uptake was persisted in mitosis (M-) phase and highly activated in DNA synthesis (S-) phase of cell-cycle. Over-expression or phosphorylation of a mitochondrial chaperone, 75-kDa glucose-regulated protein (GRP75), promoted monopolar spindle kinase 1 (MPS1)-controlled centrosome duplication and cell-cycle progress, but also driven cell-cycle-dependent macropinocytosis of Tat/pDNA-Ca^2+^ nanoparticles. Further in vivo molecular imaging based on DF (Fluc-eGFP)-TF (RFP-Rluc-HSV-ttk) system showed that Tat/pDNA-Ca^2+^ nanoparticles exhibited highly suicide gene therapy efficiency in mouse model xenografted with human ovarian cancer. Furthermore, arresting cell-cycle at S-phase markedly enhanced delivery performance of Tat/pDNA-Ca^2+^ nanoparticles, whereas targeting GRP75 reduced their macropinocytic delivery. More importantly, in vivo targeting GRP75 combined with cell-cycle or macropinocytosis inhibitors exhibited distinct suicide gene therapy efficiency. In summary, our data highlight that mitochondrial chaperone GRP75 moonlights as a biphasic driver underlying cell-cycle-dependent macropinocytosis of Tat/pDNA-Ca^2+^ nanoparticles in ovarian cancer.

**Supplementary Information:**

The online version contains supplementary material available at 10.1186/s12951-022-01530-6.

## Introduction

Current treatments of ovarian cancer (OC) mainly choose surgery and chemotherapy, resulting in limited clinical benefits [1]. Tumor-targeted suicide gene therapy is emerging as a new clinical option for OC [2]. It is based on delivery of a gene encoding enzyme that converts the nontoxic prodrug into toxic drug following administration. The cytotoxic action is localized inside tumor or, if applicable, in neighboring tumor cells that have not been transduced but undergo oncolysis due to the “bystander effect”. The most utilized suicide gene therapy is based on herpes simplex virus-thymidine kinase (HSV-TK) followed by treatment with antiviral drug ganciclovir (GCV), which is transformed to toxic metabolites by the TK action, causing failure of cancer cells in DNA replication and cell apoptosis [3]. Although suicide gene therapy is in progress, it is far from reaching OC patients. Major limitations are lacking translatable gene delivery vectors.

Introduction of cell-penetrating peptides (CPPs), typically HIV-Tat-derived peptide (Tat), to noncovalently complex therapeutic gene incorporated in plasmid DNA (pDNA) for intracellular delivery represents an innovative approach for targeting malignancy [4]. Noncovalent complexation relies on electrostatic interaction between cationic Tat and anionic pDNA, which leads to formation of large Tat/pDNA complexes with increased delivery efficiency [5, 6]. Furthermore, hydrophobic interactions between excess Tat peptides or combining with additional cationic polymers, lipids or surfactants intensively condense Tat/pDNA complexes into smaller nanoparticles with better gene delivery performance [7, 8]. Unfortunately, these Tat-based nonviral nanoparticles with high positive charge have some weaknesses, such as prone to aggregation and cytotoxicity [4, 9]. Therefore, seeking new strategies for safe and efficient delivery of Tat/pDNA complexes is required.

As a stabilizer of DNA-carrier complexes, divalent Ca^2+^ has been widely used in therapeutic gene delivery [10]. When used for a standalone gene vector, Ca^2+^ precipitates with pDNA to form condensed particles amenable to cellular transfection. Previous studies revealed that Ca^2+^ has a synergic effect on DNA condensation along with other kinds of CCPs [11–13]. This synergic effect might be attributed to the “soft” condensation ability of Ca^2+^ to DNA-complexes. Theoretically, a strong affinity between Tat and pDNA is required to form condensed and stable particles for yielding efficient uptake. On the other hand, a relatively lower affinity between Tat and pDNA is desired to facilitate pDNA release after endocytosis [10]. Because ionic strength affects the size and binding balance within charged nano-complexes [11], optimal Ca^2+^ addition controlling the delicate formulation of Tat/pDNA nanoparticles should be explored.

Macropinocytosis is an evolutionarily conserved endocytic route that mediates non-specific fluid-phase uptake of extracellular particles and solutes. It is now well appreciated that active macropinocytosis is a hallmark of certain tumors, and macropinocytosis is markedly upregulated by various oncogenic pathways or nutrient-deficient microenvironments [14, 15]. Complexation of cationic CPPs with anionic pDNA could bring some degree of endosome escape capability, but also induce macropinocytosis in targeted cells for their uptake [16, 17]. This largely attributes to CPPs-induced vesicle budding and macropinosome leakage effect [18, 19]. Thus manipulation of macropinocytosis may offer a unique opportunity to achieve tumor-targeted delivery of Tat/pDNA complexes [6, 7]. Stimulating macropinocytic uptake, blocking macropinocytic recycling and preventing fusion of macropinosomes with lysosomes are leading-edge exploration for efficient delivery of Tat-based non-viral nanoparticles [16, 17, 20, 21]. Unlike the viral nanoparticles that are transduced throughout all stages of cell-cycle, a major drawback of non-viral nanoparticles is that their gene delivery efficiency is significantly correlated with cell-cycle status [22–25]. We previously found that uptake of several nanoparticles was varied throughout cell-cycle, and a mitochondrial chaperone, 75-kDa glucose-regulated protein (GRP75), moonlights as a cell-cycle controller and endocytosis regulator in HeLa and Cos-7 cells [24, 26]. The moonlighting function of GRP75 in cell-cycle-dependent endocytosis was utilized to enhance intracellular delivery of nanomicrospheres through its targeting-induced G1-phase retention effect [24]. This attributed much to its inhibition on clathrin-mediated endocytosis (CME) but promotion on clathrin-independent endocytosis (CIE) [24, 27, 28]. Given that macropinocytosis is a key CIE pathway for the uptake of Tat-based non-viral nanoparticles [7, 14, 16, 21, 29], and Tat-compacted pDNA complexes mainly use macropinocytosis for uptake in malignancies [6, 7], investigating whether macropinocytic uptake of nanoparticles was varied during the cell-cycle of OC cells, exploiting whether GRP75-interuption affected cell-cycle-dependent macropinocytosis, and capitalizing such feature for highly efficient suicide gene therapy should increase our understanding for development of tumor-targeted suicide gene therapy.

In this study, pDNA was complexed with Tat peptide and condensed into nanoparticles by addition of calcium chloride (CaCI_2_). This simple formulation (Tat/pDNA-Ca^2+^ nanoparticles) exhibited advantages, including smaller size, positive charge, safer and highly suicide gene therapy efficiency. The Tat/pDNA-Ca^2+^ nanoparticles was delivered by cell-cycle-dependent macropinocytosis in OC cells, and their uptake was driven by expression or phosphorylation of moonlighting chaperone GRP75. Targeting GRP75 blocked monopolar spindle kinase 1 (MPS1)-controlled centrosome duplication and cell-cycle progress, but also reduced the active macropinocytosis of Tat/pDNA-Ca^2+^ nanoparticles at cell-cycle mitosis (M-) and DNA synthesis (S-) phases. In vivo targeting GRP75 combined with cell-cycle or macropinocytosis inhibitors underlies distinct therapy effect in mouse model xenografted with human ovarian cancer.

## Results and discussion

### Calcium condensation of Tat/pDNA complexes into nontoxic delivery nanoparticles

**Fig. 1:**
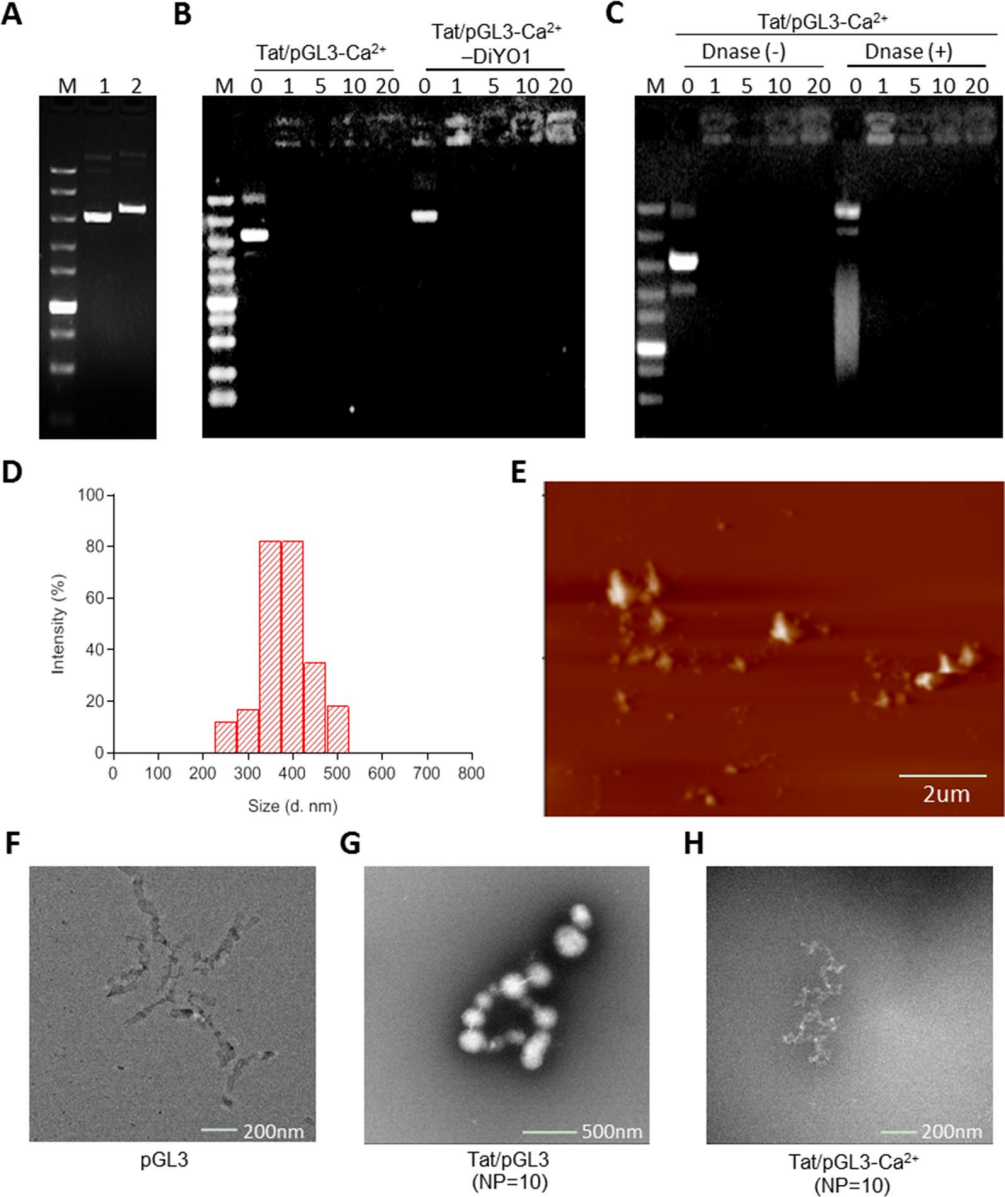
Calcium chloride facilitates the condensation of plasmid DNA with HIV-Tat-derived peptide into nanoparticles. (**A**) Agarose electrophoretic mobility assay. pDNA (pGL3 plasmid) were labeled with (2) or without (1) DiYO1. Labeling ratio was 1:5 (D: BP). M: DNA ladder. (**B**) Gel retardation assay. Lanes 2–6: Tat/pGL3-Ca^2+^ nanoparticles; lanes 7–11: Tat/pGL3-Ca^2+^-DiYO1 nanoparticles; N/P ratios between Tat and pGL3 were 0, 1, 5, 10, 20, respectively. (**C**) DNase I protection assay. Tat/pGL3-Ca^2+^ nanoparticles treated with (lanes 7–11) or without (lanes 2–6) DNase I. N/P = 0, 1, 5, 10, 20, respectively. (**D**) Size distribution of Tat/pGL3-Ca^2+^ nanoparticles determined by DLS. N/P = 10. (**E**) AFM image of Tat/pGL3-Ca^2+^ nanoparticles. Scale bar: 1 um. (**F, G, H**) TEM imaging of pGL3, Tat/pGL3 and Tat/pGL3-Ca^2+^ nanoparticles negatively stained with uranyl acetate. N/P = 10

To reduce the toxicity and at same time to improve the gene delivery efficiency of Tat/pDNA complex, calcium chloride was used as a compact agent to condense the particles of Tat/pGL3 complex. Gel retardation assay shown that Ca^2+^ addition (final conc. 113 mM) efficiently condensed Tat/pGL3 or Tat/pGL3-DiYO-1 complexes into nanoparticles even when the N/P ratio reached to 1 (Fig. 1A, B). This is a significant improvement from our previous observation that Tat-facilitated pDNA compact formation only occurred when N/P ratios more than 5 [6]. Condensation ability of Ca^2+^ addition was consistent when the N/P ratio of Tat/pDNA complexes increased from 1 to 20. Condensing stability of Tat/pDNA-Ca^2+^ nanoparticles was determined by DNase I protection assay. Total resistance to DNase I action was observed for Tat/pGL3-Ca^2+^ nanoparticles induced at N/P ratios not less than 1 (Fig. 1C). DLS (dynamic light scattering) measurements showed that formulated Tat/pGL3-Ca^2+^ nanoparticles presented weak positive ζ potential (average 3.8 mV), and possessed small particle size (252–512 nm) at N/P ratio of 10 (Fig. 1D. Additional file 1: Table S1. Additional file 2: Fig. S1). These results are similar to previous findings [11, 30], highlighting the effect of Ca^2+^ addition to Tat/pDNA complexes induced substantial decrease in the particle size and competitively inhibit the amine/phosphate interaction. This synergic effect might be attributed to the “soft” condensation ability of Ca^2+^ to Tat/pDNA complexes, because defined concentration range of calcium interactions with both amines (polycations) and phosphates (DNA) can control particle size [11, 31, 32]. The uneven size-distribution of Tat/pGL3-Ca^2+^ nanoparticles is compatible with the results from AFM and TEM checking images, and irregular granules with asymmetric morphology were frequently observed (Fig. 1E–H). High positive ζ potential and small diameter of Tat/pGL3-Ca^2+^ nanoparticles were reached with enhanced N/P ratios (Additional file 1: Table S1). Without Ca^2+^ addition, we only observed big, loose complexes between Tat peptide and pDNA [6], whereas small, compact Tat/pDNA nanoparticles were formed after Ca^2+^ addition (Fig. 1E–H. Additional file 1: Table S1).

**Fig. 2:**
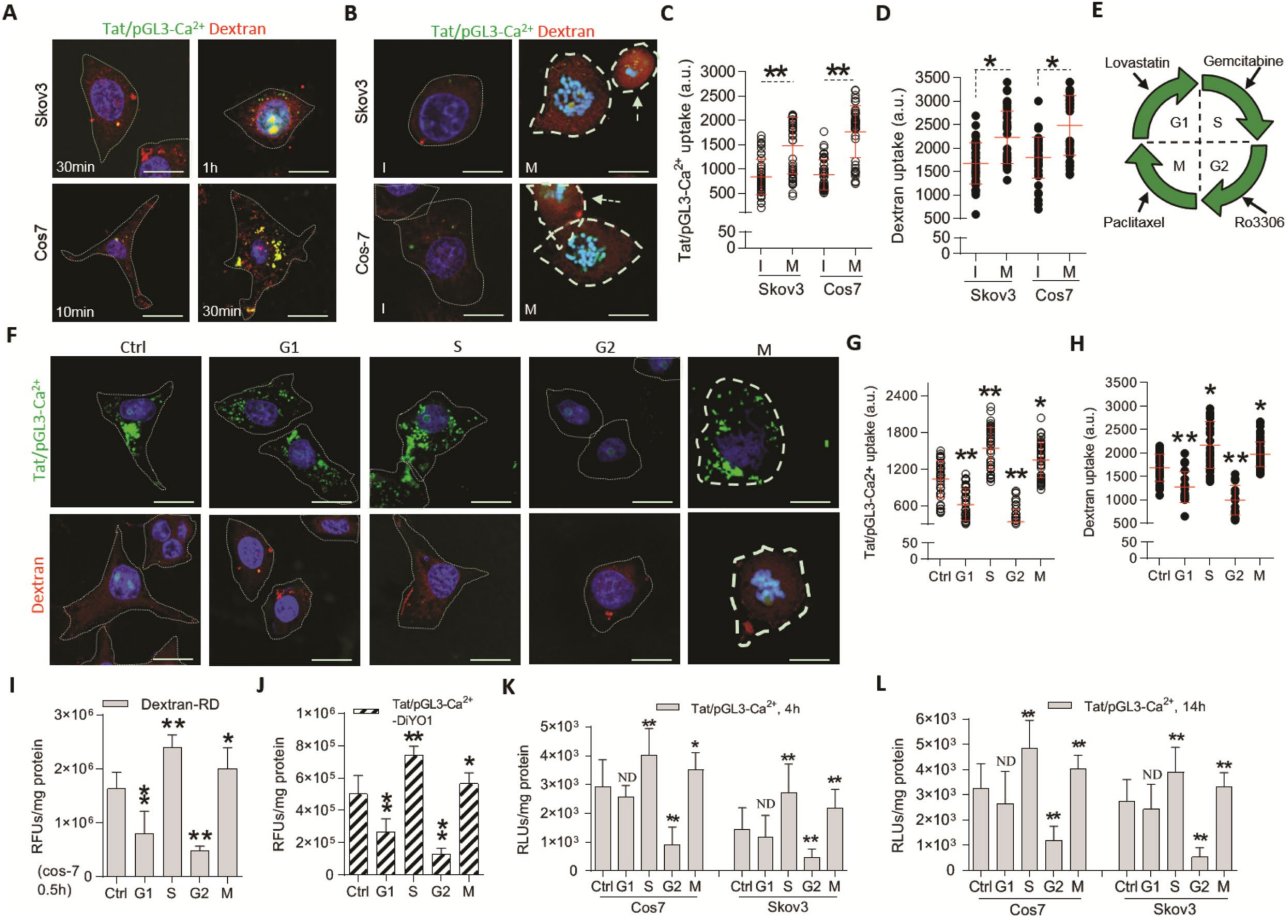
Cell-cycle-dependent macropinocytosis and expression of Tat/pDNA-Ca^2+^ nanoparticles. Confocal imaging the uptake of Tat/pGL3-Ca^2+^-DiYO1 nanoparticles mixed with Dextran-RD in Skov3 and Cos7 cells: **(A)** uptake for different time, (**B)** uptake in cell-cycle of I- (polygons with thin dotted line) and M-phases (polygons with bold solid line), (**F)** uptake in sub-phases of cell-cycle (Cos7, 4 h). Cell nuclei were stained with Hoest33342. Scale bar: 10 μm. (**C**, **D**, **G**, **H)** Scatterplots depict the uptake level of particles in the indicated cell population. 60 cells were counted for any sub-phase in each experiment. (**E)** Sub-phase-arrested cells were induced by drug treatments as described in ‘Materials and Methods’. (**I**, **J)** Uptake level of Dextran-RD and Tat/pGL3-Ca^2+^-DiYO1 in sub-phase-arrested Skov3 cells was quantified by fluorescence microplate reader. RFUs/mg protein: relative fluorescence intensities per milligram protein. **(K**, **L)** Luciferase activity of sub-phase-arrested cells transduced with Tat/pGL3-Ca^2+^ nanoparticles for 4 and 14 h, respectively. RLUs/mg protein: relative light units of expressed luciferase per milligram. n = 3. Statistically significant differences in relation to control (no drug treatment) are shown: **P < 0.01, *P < 0.05

Compared to previous formulation [6, 7], adding Ca^2+^ in this work led to high-efficient delivery of Tat/pDNA complexes without apparent toxicity (Figs. 2, 7E, Additional file 2: Fig. S2, S13). Less apoptosis-induced in OC cells and negligible body weight or visceral changes in OC-bearing mice were observed after treatment with Tat/pDNA-Ca^2+^ nanoparticles. Noticeably, improved cell survival was observed after adding Ca^2+^ into Tat/pDNA complex (Additional file 2: Fig. S2C-D), and only high-concentration (20ug/mL, NP = 10), long-term (14 h) exposure of Tat/pGL3-Ca^2+^ nanoparticles to cells can trigger necrotic apoptosis (Additional file 2: Fig. S2A-B). It was reported that Ca^2+^ addition (> 30 mM) can reduce aggregation and yield of more monodisperse lipoplexes [33]. We observed agglomeration of Tat/pDNA complexes without CaCl_2_, whereas Tat/pDNA-Ca^2+^ nanoparticles with optimal CaCl_2_ concentration (113 mM) were relatively stable in mouse serum and serum-contained culture media (Additional file 2: Fig. S3). This stability was also demonstrated by the heparin displacement assay, because exposing the Tat/(SYBR Green)-pDNA-Ca^2+^ nanoparticles to the highly negatively charged heparin yielded an increase in the fluorescence signal with increase in heparin concentration (Additional file 2: Fig. S4). Stable body weight of mice and no visceral toxicity in OC-bearing mice (Additional file 2: Fig. S13, Fig. 7E), indicate that the formulated Tat/pDNA-Ca^2+^ nanoparticles are safe. These results demonstrated that Tat/pDNA complexes were tightly condensed into small, nontoxic delivery nanoparticles by Ca^2+^ addition.

### Cell-cycle-dependent macropinocytic uptake of Tat/pDNA-Ca^2+^ nanoparticles

Macropinocytosis was reported as a dominant uptake route for Tat-based gene delivery vectors [6, 7, 16, 34–36]. Here, we found that Tat/pGL3-Ca^2+^ nanoparticles mainly utilized macropinocytosis for uptake in OC cells. Using various endocytic markers as indicated in the literatures, confocal imaging showed that Tat/pGL3-Ca^2+^ nanoparticles were not co-localized with the clathrin-mediated endocytosis (CME) marker Tfn-AF647 and lipid raft marker CTxB, but co-localized with the macropinocytosis marker Dextran (Fig. 2A, Additional file 2: Fig. S5A, S6). SKOV3 cells pretreated with CME inhibitor CPZ, or caveolae-mediated endocytosis (CvME) inhibitor filipin, or cholesterol-depleting reagent MBCD did not significantly inhibit the uptake of Tat/pGL3-Ca^2+^ nanoparticles. In contrast, when cells were pretreated either with the macropinocytosis inhibitor EIPA or with CyD, the uptake of nanoparticles was significantly inhibited (Additional file 2: Fig. S5B). These results were similar to our previous observations [6, 7, 27]. The phenomenon that Tat/pDNA-Ca^2+^ nanoparticles preferred macropinocytic uptake in OC cells can be explained by: (1) since macropinocytic capacity is determined by the properties of cells [14, 15], intensified macropinocytosis of Tat/pDNA complex was induced in OC cells with certain mutations [27]. (2) Positive-charged, botryoid-shaped nanoparticles enter tumor cells via macropinocytosis more efficiently than negative-charged, shape-defined nanoparticles [17, 37]. Our Tat/pDNA-Ca^2+^ nanoparticles with irregular and asymmetric morphology (Fig. 1E, H) largely manipulate macropinocytosis to favor their intracellular delivery; (3) Cationic CPPs binding with anionic glycosaminoglycans (GAGs) on cell surface always stimulate Rac activation, which induces actin polymerization, lamellipodia formation and subsequent macropinocytosis-initiation [20, 38, 39]. Excess Tat peptide within Tat/pDNA-Ca^2+^ nanoparticle (NP > 10) are prone to stimulate macropinocytosis boosted in OC cells (Fig. 2, 4); (4) Cancer cells undergoing blebbishield emergency program exhibit robust macropinocytosis [40], the pro-apoptotic status of OC cells stimulated by Tat/pDNA-Ca^2+^ nanoparticles (Additional file 2: Fig. S2) may further promote the macropinocytosis in these cells. In addition, we also observed macropinocytic difference between Skov3 and Cos7 cells (Fig. 2K, L, Fig. 4A–D), suggesting the cell-specific uptake of Tat/pDNA-Ca^2+^ nanoparticles [4, 32], which is of importance for design treatment of different cell types.

Different from the prevailing claims that pinocytosis is shut down during mitosis [41–43], we found here that macropinocytosis was decreased but not completely arrested in cell-cycle M-phase. Confocal microscopic examining the co-uptake of Tat/pGL3-Ca^2+^ nanoparticles with Dextran in unperturbed cells showed that they were still internalized via macropinocytosis in different sub-phases of cell-cycle (Fig. 2A–H). Macropinocytic uptake of Tat/pGL3-Ca^2+^ nanoparticles appeared frequently shutdown in cells at I-phase (interphase) (Fig. 2B–D). To clarify these variations, we synchronized cells at distinct sub-phases of cell-cycle by chemotherapeutics pretreatments (Fig. 2E). Confocal microscopic examining the co-uptake of Tat/pGL3-Ca^2+^ nanoparticles with Dextran in arrested cell-cycle phases showed that their macropinocytosis were markedly increased in GCB(gemcitabine)-induced S-phase and persisted in PTX(paclitaxel)-induced M-phase (Fig. 2F–H). Determining these variations by fluorescence quantification assay also confirmed the enhanced co-uptake of Tat/pGL3-Ca^2+^ nanoparticles with Dextran in cells at S- and M-phases (Fig. 2I, J). Addition of macropinocytosis inhibitor EIPA (5-(N-ethyl-N-isopropyi)-amiloride) totally reversed this enhancement (Additional file 2: Fig. S6A, B). Further checking the impact of Tat/pDNA-Ca^2+^ nanoparticles on cell-cycle distribution found that sub-phases of asynchronous or synchronized cells were not significantly altered after incubating with Tat/pGL3-Ca^2+^ nanoparticles (Additional file 2: Fig. S7). Additionally, enhanced transgene expression of Tat/pGL3-Ca^2+^ nanoparticles in S- or M-phase cells and transduction-inhibition by EIPA co-incubation were observed (Fig. 2K, L, Additional file 2: Fig. S6C). These results suggest that macropinocytosis and expression of Tat/pDNA-Ca^2+^ nanoparticles were persisted in cell-cycle M-phase and markedly increased in cell-cycle S-phase.

**Fig. 3:**
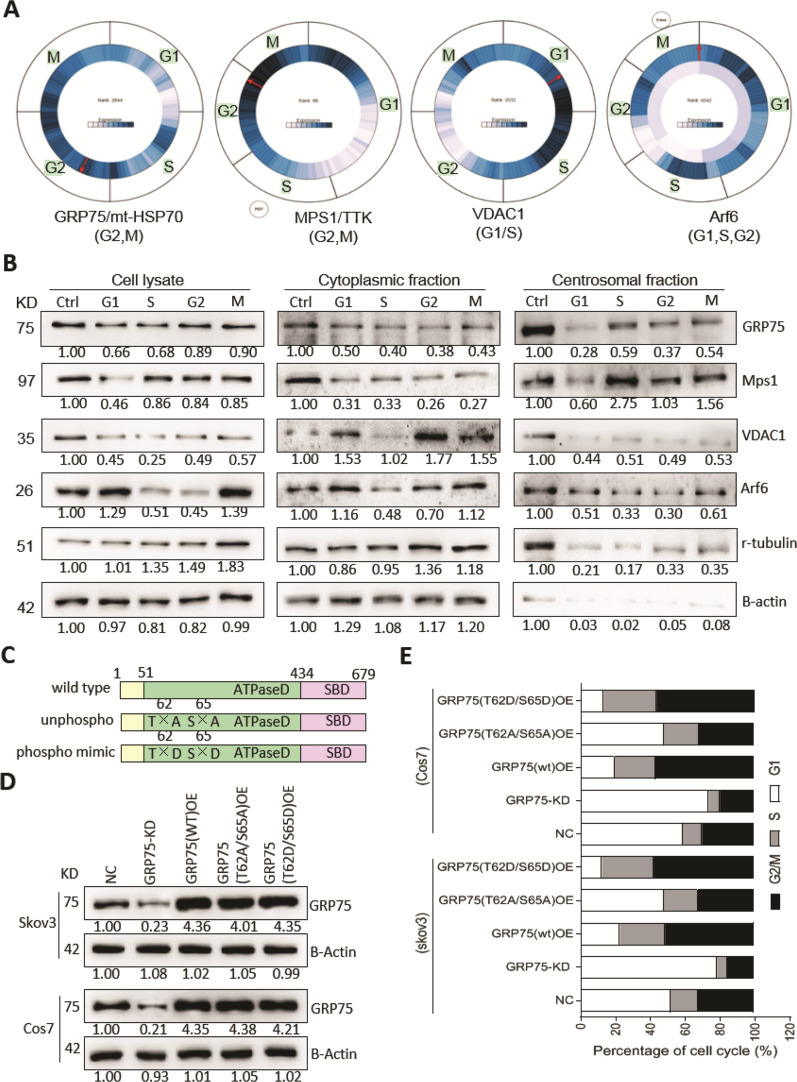
Cell-cycle-dependent centrosomal enrichment of GRP75 and its phosphorylation acting on sub-phase distribution. **(A)** Cyclebase-based predication of expression of GRP75, MPS1, VDAC1 and Arf6 in sub-phases of cell-cycle (dark blue content indexes the mRNA level during cell-cycle). **(B)** Western blot determined the expression level of those mentioned protein in subcellular fractions of synchronized Skov3. Protein bands were quantified by Image J software and expression ratio (compared to cells without drug-induction, Ctrl, set as 1) in the sub-phase are correspondingly marked below. **(C)** Schematic GRP75 constructs: wt: wild type. unphospho: unphosphorlation (T62A/S65A). phospho mimic: phosphorlation mimic (T62D/S65D). **(D**) Lentivirus-stable-transduced Skov3 and Cos7 cells were built based on GRP75-targeting shRNA (GRP75-KD), or over-expression (OE) with constructs as listed above. GRP75 expression level was analyzed by western blotting. (**E**) Bar graphs show the sub-phase percentage of cell-cycle in GRP75-KD or -OE cells. Data are the average results of three independent experiments

Previous studies showed that several cellular uptake pathways (caveolar and clathrin-mediated endocytosis) do take place during normal mitosis when checking asynchronous cells under advanced microscopy (e.g. Electron microscopy) [44–46]. Considering only 0.5–2% of mammalian cells undergoing mitosis and residual endocytosis are dedicated to the turnover of plasma membrane and specific receptors during the successive process (prophase, metaphase, anaphase, telophase and cytokinesis) [42, 47], re-activated macropinocytosis of Tat/pGL3-Ca^2+^ nanoparticles in partial cell population at M-phase are logical (Fig. 2B–H). Additionally, upregulated expression of macropinocytosis driver Arf6 at M-phase (Fig. 3B) also suggests that reactivated macropinocytosis is liable to support plasma membrane remodeling when cells undergoing division. Notably, the finding here is that macropinocytosis of Tat/pGL3-Ca^2+^ nanoparticles was highly upregulated in GCB-induced S-phase cells (Fig. 2B–H). To our knowledge, this is the first observation that macropinocytosis peaked up at specific phases of cell-cycle. In vivo data with enhanced delivery and killing efficiency of Tat/TF-Ca^2+^ nanoparticles in GCB-administrated group also confirmed this finding (Figs. 7C, D, 8A, B). Little is known about the factors accounting for this contrast phenomenon. As intact nuclear membrane and undersized nuclear pore complex (10–26 nm) are impossible to assist the nuclear uptake of Tat/pDNA-Ca^2+^ nanoparticles when transfection was carried out at this phase, possibly factors may be from certain stages of macropinocytosis. High macropinocytosis of Tat/pDNA-Ca^2+^ nanoparticles but low Arf6 expression at S-phase appears paradoxical (Fig. 2F–H, Fig. 3B). Since Arf6 can regulate macropinocytosis and also act on the terminal stage of cytokinesis [48, 49], highly expression and accumulation of Arf6 at M-phase is beneficial for its localizing at cleavage furrow and midbody so as to finish the cytokinesis process. Although activated Arf6 (GTP binding) is required for cell surface recycling of short-lived macropinosome, inactivation of Arf6 (GTP hydrolysis) is essential to intracellular macropinosome trafficking [31, 50], therefore, low expression of Arf6 at S-phase contributes to active macropinocytosis is logical. In addition, Tat-based nanoparticle bound with cell surface GAGs is an essential prerequisite for their uptake [38]. Because the structural and quantitative differences of GAGs (especially on the cell surface) during cell-cycle are still debated [33, 51], it’s hard to relate macropinocytosis kinetics to the expression profile of GAGs in the cells, yet.

### GRP75 driven cell-cycle-dependent macropinocytosis of Tat/pDNA-Ca^2+^ nanoparticles

Seeing that cell-cycle and endocytosis-transition are synchronously regulated by moonlighting chaperones [24, 52, 53], we next determined whether mitochondrial moonlighting chaperone GRP75 acting on the cell-cycle-dependent macropinocytosis of Tat/pDNA-Ca^2+^ nanoparticles. Cyclebase-based predication showed that high-expression of GRP75 frequently appeared at S-phase and G2(gap phase 2)/M boundary. High-expression of GRP75-bound mitotic kinase MPS1 was mainly distributed from S- to M-phase. However, high-expression of GRP75-bound, MPS1-recruited mitochondrial gatekeeper VDAC1 was mainly presented at S-phase. Remarkably, high-expression of macropinocytosis driver Arf6 was scarcely distributed at S-phase, G2/M boundary and early G1-phase (gap phase 1) (Fig. 3A). Western blot analysis of cell fractions showed that centrosome-associated GRP75 and MPS1 were highly enriched at S-, G2-, and M-phases than that at G1-phase. In contrast, centrosome-associated VDAC1 and Arf6 were sharply reduced at these phases (Fig. 3B). Unexpectedly, whole lysate-derived and cytoplasmic VDAC1, Arf6 were reduced at S-phases, and no significant expression-difference of GRP75 and MPS1 was detected. These results suggest that GRP75 and MPS1 co-enriched at centrosome from cell-cycle S- to M-phase.

**Fig. 4:**
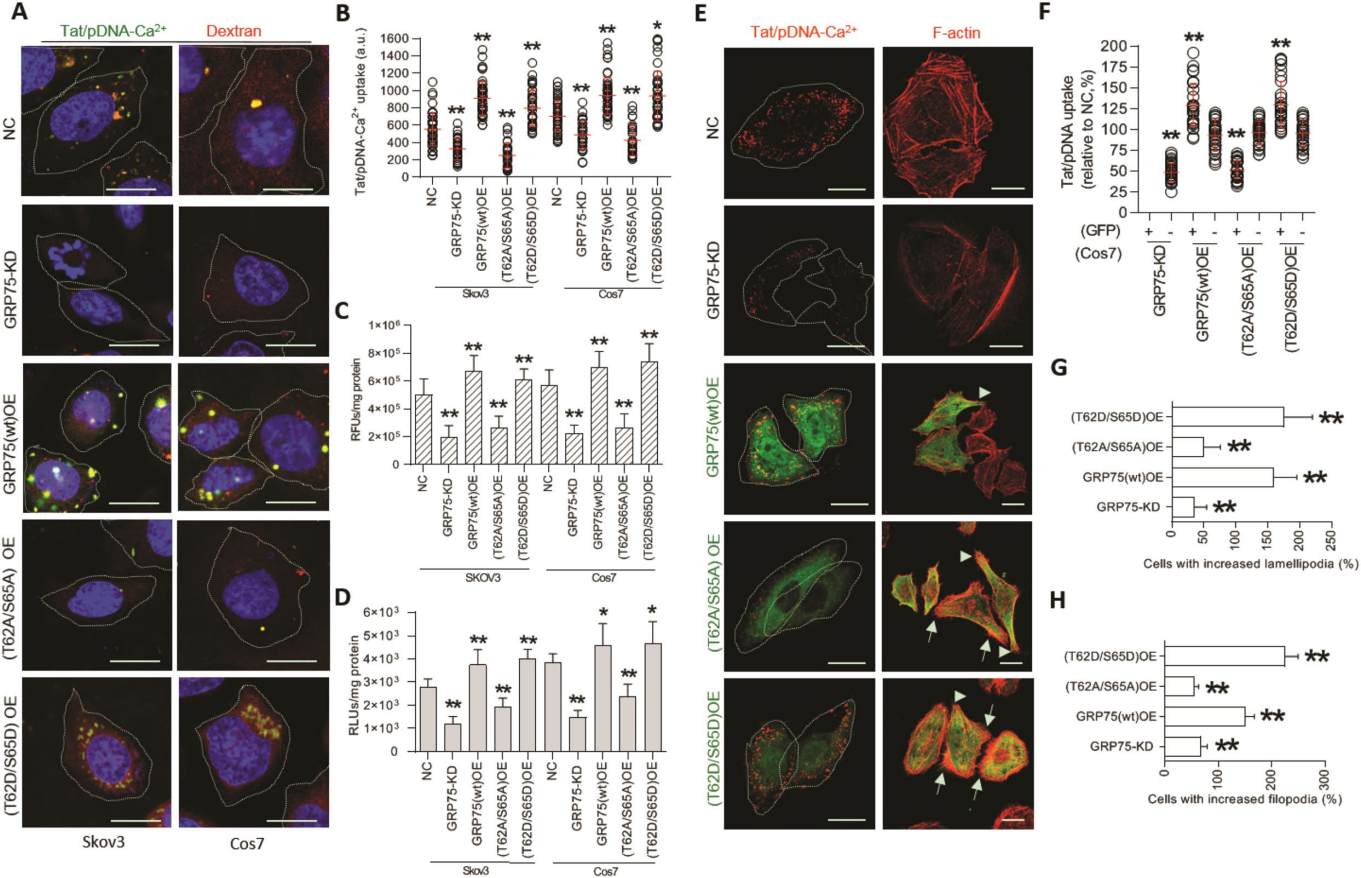
Highly expression or phosphorylated activation of GRP75 promotes the macropinocytosis of Tat/pGL3-Ca^2+^ nanoparticles. (**A)** Confocal imaging the co-uptake of Tat/pGL3-Ca^2+^-DiYO1 nanoparticles and dextran-RD (1 h) in cells with GRP75-KD or -OE. Scale bar: 10 μm. **(B)** Scatterplots depict the uptake level of Tat/pGL3-Ca^2+^ nanoparticles in single cell population with GRP75-KD or -OE. **(C)** Uptake quantitation of Tat/pGL3-Ca^2+^-DiYO1 in GRP75-KD or -OE cell population by the fluorescence microplate reader. (**D)** Luciferase activity of GRP75-KD or -OE cells transduced with Tat/pGL3-Ca^2+^ nanoparticles for 14 h. **(E)** Confocal imaging the uptake of Tat/pGL3-Ca^2+^-DiYO3 nanoparticles in Skov3 cells with GRP75-KD or transfected with EGFP-fused GRP75 constructs. Polymerized actin fibers were detected by staining with Rhodamine-phalloidin. Scale bar, 10 μm. (**F)** Scatterplots depict the uptake level of Tat/pGL3-Ca^2+^-DiYO3 nanoparticles in indicated cells. **(G–H**) Morphometric analyses of the lamellipodia and filopodia formation in indicated cells. > 60 cells were counted for each cell line or transfection. n = 3. Statistically significant differences in relation to NC (negative control) group are shown: **P < 0.01, *P < 0.05

GRP75 was shown being associated with duplicated centrosomes, phosphorylated by Mps1 on Thr62 and Ser65, and feedback super-activated Mps1 in HeLa-1 and U2OS cells [54, 55]. To determine whether it could moonlight as a regulator on cell-cycle and macropinocytosis, we created lentivirus stable-transfected Cos7 and Skov3 cells with GRP75-knock-down (KD) and –over-expression (OE) of its phosphorylation mutants (Fig. 3C, D, Additional file 2: Fig. S8). Flow cytometry analysis showed that GRP75-KD or phosphorylation-inactivation (T62A/S65A) significantly induced cell-cycle accumulated at G1-phase. In contrast, GRP75-OE or phosphorylation-activation (T62D/S65D) promoted cell-cycle S-phase enriched and M-phase arrested (Fig. 3E). Confocal checking the uptake of Tat/pGL3-Ca^2+^ nanoparticles showed that their macropinocytosis were markedly reduced in GRP75-KD or phosphorylation-inactivation (T62A/S65A) cells, but significantly enhanced in GRP75-OE or phosphorylation-activation (T62D/S65D) cells (Fig. 4A, B). Such distinct uptake of Tat/pGL3-Ca^2+^ nanoparticles in GRP75-interrupted cells was also observed in fluorescence quantification assays (Fig. 4C), and correspondingly resulted in the variation of transgene expression (Fig. 4D). Additionally, distinct macropinocytosis of Tat/pGL3-Ca^2+^ nanoparticles were observed in EGFP-positive, GRP75-transiently-transfection cells (Fig. 4E, F). These results suggest that cell-cycle-dependent macropinocytosis of Tat/pDNA-Ca^2+^ nanoparticle was driven by high-expression or phosphorylation of GRP75.

Although further work is needed to deeply dissect why macropinocytosis was highly upregulated by GRP75 and its phosphorylation (Fig. 4), the potential possibilities can be: (1) GRP75-OE increase the expression of S-phase transcription factor E2F-1 and cyclin-dependent kinase inhibitor p21 [47]. P21-activated kinase 1 (Pak-1) can promote CPPs uptake and macropinocytosis [14]; (2) GRP75 interacting with dynein light chain is involved in membrane-associated trafficking [41]. As a Pak1-interacting substrate, dynein light chain phosphorylation controls macropinocytosis [54]. Therefore, phosphorylation-modified GRP75 may significantly modulate this process; (3) we have demonstrated that GRP75-KD or inhibition significantly reduced Rac1 activation [34]. Since Rac1 activation always induces membrane ruffling and macropinocytic cup formation [32], dynamic expression and phosphorylation of GRP75 from S- to M-phase modulate macropinocytosis is logical (Fig. 8E).

### GRP75 promoted centrosome duplication via recruiting Mps1 to centrosome

**Fig. 5:**
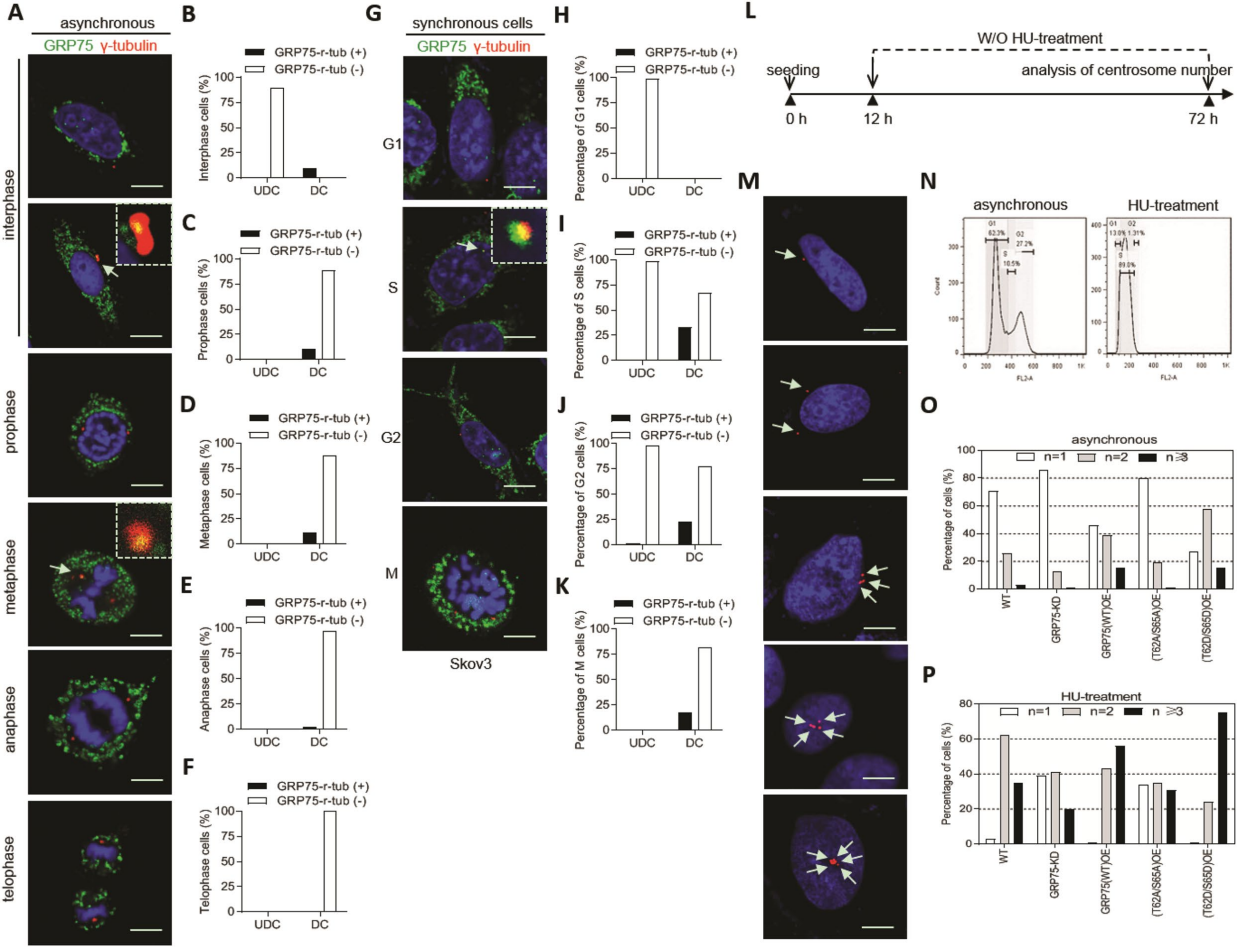
Highly expression or phosphorylated activation of GRP75 promotes centrosome duplication. **(A**, **G**) Confocal imaging the co-localization of GRP75 with γ-tubulin in asynchronously and synchronously growing Skov3 cells. Arrows point to the positions of centrosomes. Images placed on the up-right show the magnification of areas indicated by arrows. Scale bar, 10 μm. **(B**–**F**, **H**–**K**) The frequencies of GRP75 co-localization with γ-tubulin among unduplicated and duplicated centrosomes were determined and plotted, respectively. (**L**) Schematic centrosome reduplication assay. (**M**) Confocal imaging of replicated and non-replicated centrosomes in hydroxyurea (HU)-exposed cells, and **(N)** cell-cycle was determined by flow cytometry analysis. (**O**, **P**) The frequencies of centrosomes per cell were scored and shown in asynchronously and synchronously growing Skov3 cells with GRP75-KD or -OE. 60 cells were counted for any sub-phase or cell line. Average values are shown

Dual-specific protein kinase MPS1 controls a number of steps in cell-cycle. Its activation promotes centrosome duplication but inhibits mitotic checkpoint response [56]. The finding of co-enrichment of MPS1 with GRP75 during cell-cycle (Fig. 3A, B) promoted us to explore whether centrosome duplication was regulated by GRP75. Confocal checking the asynchronously growing Cos7 and Skov3 cells showed that GRP75 did not bind with unduplicated centrosomes during cell-cycle, but bound with partial fraction of duplicated centrosomes during cell-cycle (Fig. 5A-F, Additional file 2: Fig. S9A-F). Next, the association of GRP75 with duplicated centrosomes from S- to M-phase in synchronously growing cells was observed by microscopy examination (Fig. 5G–K, Additional file 2: Fig. S9G-K). Since total expression level of GRP75 through the cell-cycle was at a similar level (Fig. 3B), phase-dependent association of GRP75 with duplicated centrosomes promoted us to further explore whether its expression or modification affects centrosome duplication. In hydroxyurea (HU)-induced centrosome re-duplication assays (Fig. 5L–N, Additional file 2: Fig. S9L-M), GRP75-OE significantly increased the frequency of centrosome amplification, and GRP75-KD markedly reduced the centrosome amplification. Notably, more increased centrosome amplification was detected in GRP75 phosphorylation-activation (T62D/S65D) cells, while less centrosome amplification was found in GRP75 phosphorylation-inactivation (T62A/S65A) cells (Fig. 5P, Additional file 2: Fig. S9O). Similar changes of centrosome amplification were found in asynchronously growing cells upon modulation of GRP75-expression level (Fig. 5O, Additional file 2: Fig. S9N). These results suggest that high-expression or phosphorylated activation of GRP75 is required for centrosome duplication.

**Fig. 6:**
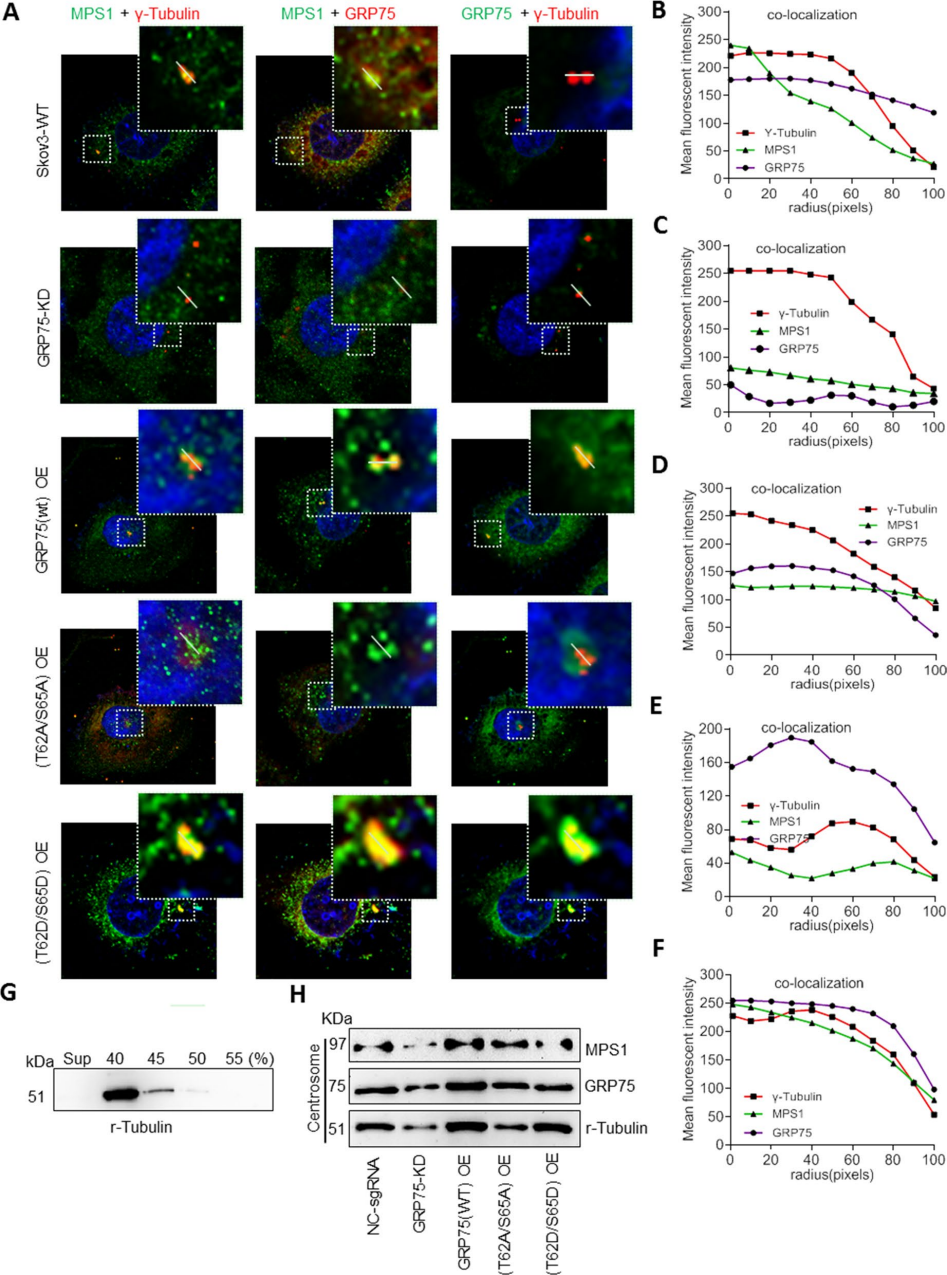
Highly expression or phosphorylated activation of GRP75 promotes itself and Mps1 translocating to centrosome. **(A)** Confocal imaging the co-localization of MPS1 and γ-tubulin, GRP75 and γ-tubulin, GRP75 and γ-tubulin in Skov3 cells with GRP75-KD or -OE. **(B**–**F)** Fluorescent intensities along the central line of centrosome were plotted, and staining signal distribution curves of GRP75, MPS1 and γ-tubulin were determined by ImageJ software and are shown. **(G)** Centrosome extracts prepared from Skov3 cells were subjected to sucrose gradient centrifugation, and the fractions were western blotted to identify the fraction enriched for centrosomes. **(H)** Western blot determined the centrosome-resident level of GRP75 and MPS1 in Skov3 cells with GRP75-KD or -OE

As chaperone protein complexed with mitotic kinases can modulate their activity during the progression of cell-cycle [57, 58], we next tested whether centrosome-targeting of MPS1 was dependent on the presence of GRP75. Confocal checking the association of GRP75 and MPS1 at centrosome showed that these two targets were unable to simultaneously localize at centrosomes in GRP75-KD and phosphorylation-inactivation (T62A/S65A) cell. In contrast, significant centrosomal co-localization of GRP75 with MPS1 was detected in GRP75-OE and phosphorylation-activation (T62D/S65D) cells (Fig. 6A–F, Additional file 2: Fig. S10A-F). Further checking the centrosome-resident MPS1 in isolated fractions confirmed that GRP75-OE or phosphorylation-activation markedly increased MPS1 level, while GRP75-KD or phosphorylation-inactivation substantially reduced MPS1 level at centrosome (Fig. 6G, H, Additional file 2: Fig. S10G). These results suggest that centrosome-recruited MPS1 was promoted by high-expression or phosphorylation of GRP75.

Moonlighting protein has unrelated functions depending on the dynamic cellular context, which provides a connection between distinct biochemical processes [52, 59]. We have found that GRP75 is functionally enriched in heparan sulfate proteoglycan (HSPG)-mediated and membrane raft-associated endocytosis vesicles [26]. It can moonlight as a cell-cycle controller and endocytosis regulator [24, 27, 28]. Here, in OC cells, we also observed that GRP75-depletion induced cell-cycle G1-phase accumulation and GRP75-over-expression induced M-phase enrichment (Fig. 3E), reinforcing its role on cell-cycle control. Previous studies reported that GRP75 might control cell-cycle progress via cyclin-dependent kinas/TP53/Rb signaling [54, 60]. Our present data show that GRP75 associated preferentially with duplicating centrosomes (Fig. 5, Additional file 2: Fig.S9) and its phosphorylation was critically required for Mps1-translocating to centrosome (Fig. 6). Since MPS1 control centrosome duplication and mitotic checkpoint response and phosphorylated GRP75 super-activates Mps1 in a feedback manner [55, 56], the interdependence of GRP75 with MPS1 on centrosome-targeting (Fig. 6, Additional file 2: Figs. S10, S11) suggests that GRP75 is also required for centrosome duplication and mitotic checkpoint response. Additionally, centrosome is a highly unstable macromolecular complex and Hsp70–Hsp90 chaperone machinery is essential to maintain centrosome integrity for cell-cycle progression [57, 58]. Thus, centrosome-recruiting of GRP75 from S- to M-phase (Figs. 5, 6) may well help its maturation and accurate assembly of the bipolar spindle.

### GRP75-driven, cell-cycle-dependent macropinocytosis in nanoparticle therapy

**Fig. 7:**
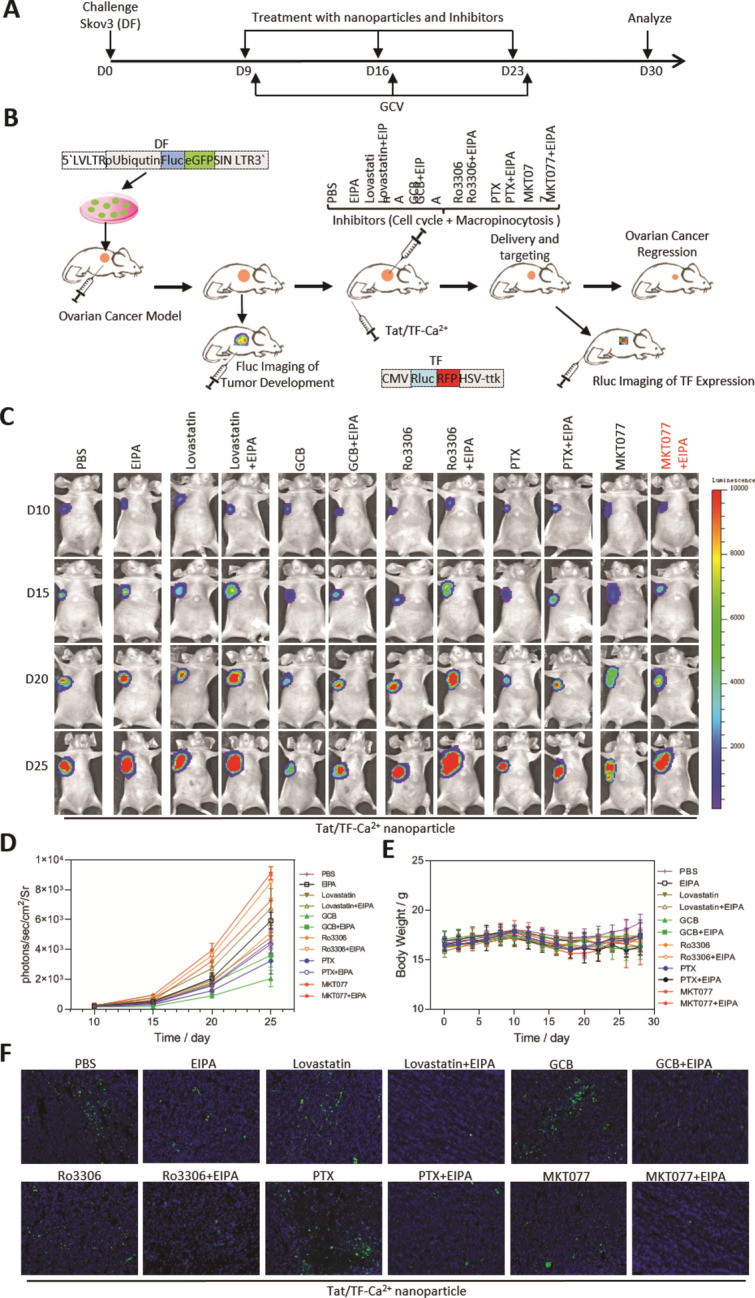
Inhibition of GRP75-driven, cell-cycle-dependent macropinocytosis attenuated Tat/TF-Ca^2+^ nanoparticles-based suicide gene therapy. Schematic model of Tat/TF-Ca^2+^ nanoparticles for in vivo imaging and suicide gene therapy. (**A)** Long-term GCV-trigged nanoparticle-based therapy in ovarian cancer model. **(B)** DF-transduced Skov3 cells were subcutaneously injected for development of ovarian cancer model, and tumor progression was tracked by Fluc imaging in vivo. Tat/TF-Ca^2+^ nanoparticles were injected via the tail vein, and delivery of nanoparticles into tumor was tracked by Rluc imaging. Regression of ovarian cancer could be reached by intratumoral administration of inhibitors of cell-cycle or macropinocytosis. Illustration of DF (Fluc-eGFP) and TF (RFP-Rluc-HSV-ttk) reporter genes were illustrated. **(C)** Fluc imaging of ovarian cancer development in vivo. Representative mouse from distinct treatments is presented: (1) PBS. (2) EIPA. (3) Lovastatin. (4) Lovastatin + EIPA. (5) GCB. (6) GCB + EIPA. (7) Ro3306. (8) Ro3306 + EIPA. (9) PTX. (10) PTX + EIPA. (11) MKT077. (12) MKT077 + EIPA. (**D**) Quantitative analysis of mouse BLI signals being shown as photons/sec/cm2/sr. **(E)** Body weight of the ovarian tumors at 21 day post-administration with Tat/TF-Ca^2+^ nanoparticles and treatments with different inhibitors (n = 6). **(F)** TUNEL-assay-determined cellular apoptosis of ovarian tumors within different groups

To explore whether GRP75-driven, cell-cycle-dependent macropinocytosis contribute to the uptake of Tat/pDNA-Ca^2+^ nanoparticles in vivo, inhibitors of GRP75, cell-cycle and macropinocytosis were applied to modulate the delivery of Tat/pDNA-Ca^2+^ nanoparticles in animals. NOD/SCID mice were subcutaneously transplanted with Skov3 cells labeled with DF (double fusion, Fluc-eGFP) reporter gene, and Tat/TF (triple fusion, RFP-Rluc-HSV-ttk)-Ca^2+^ nanoparticles were introduced by tail vein injection for suicide gene therapy (Fig. 7A, B). Bioluminescence imaging showed that, with GCV administration to induce suicidal cell-killing, Fluc signal from all groups with Tat/TF-Ca^2+^ therapy increased slightly until d15, indicating efficient tumor-suppression by the nanoparticles. When macropinocytosis or GRP75 inhibitor was intratumoral injected, Fluc signal from EIPA and MKT077 groups significantly stronger than that of the PBS group (Fig. 7C, D). In parallel groups with intratumoral injection of cell-cycle inhibitors, Fluc signal from GCB- and PTX-groups sharply declined, indicating more extensively regression of OC growth than nanoparticle mono-therapy. In contrast, significantly higher Fluc signal from lovastatin- and Ro3306-groups were observed compared to the nanoparticle mono-therapy group (Fig. 7C, D). Because cell-cycle inhibitors distinctly affected OC regression by Tat/TF-Ca^2+^ nanoparticles, we tried additionally intratumoral injection with EIPA in the four test groups. Comparing to the increased lovastatin- or Ro3306-induced Fluc signal, combined treatment with EIPA further markedly enhanced the Fluc signal, indicating decreased nanoparticle-therapy effect, due to the reduced macropinocytosis at cell-cycle G1- or G2-phase. Notably, combining EIPA with GCB or PTX brought reversed Fluc signal when compared to mono-treatment with GCB or PTX, indicating enhanced nanoparticle-therapy effect at S- or M-phase was due to active macropinocytosis at these two cell-cycle sub-phases (Fig. 7C, D). Furthermore, when MKT077 and EIPA were combined for intratumoral injection, the therapy effect of Tat/pDNA-Ca^2+^ nanoparticles was largely attenuated (Fig. 7C, D). We also checked body weight of treated mice, and no significant changes were observed in all experimental animals (Fig. 7E). TUNEL assays showed that treatment with Tat/TF-Ca^2+^ nanoparticle alone induced markedly apoptosis in OC tissues. More significant apoptosis was detected in the group with additional GCB- and PTX-treatments, and increased apoptosis was eliminated when EIPA was co-applied (Fig. 7F, Additional file 2: Fig. S12). These data strongly suggest that Tat/TF-Ca^2+^ nanoparticle-based suicide gene therapies were dominantly controlled by GRP75-driven, cell-cycle-dependent macropinocytosis.

**Fig. 8:**
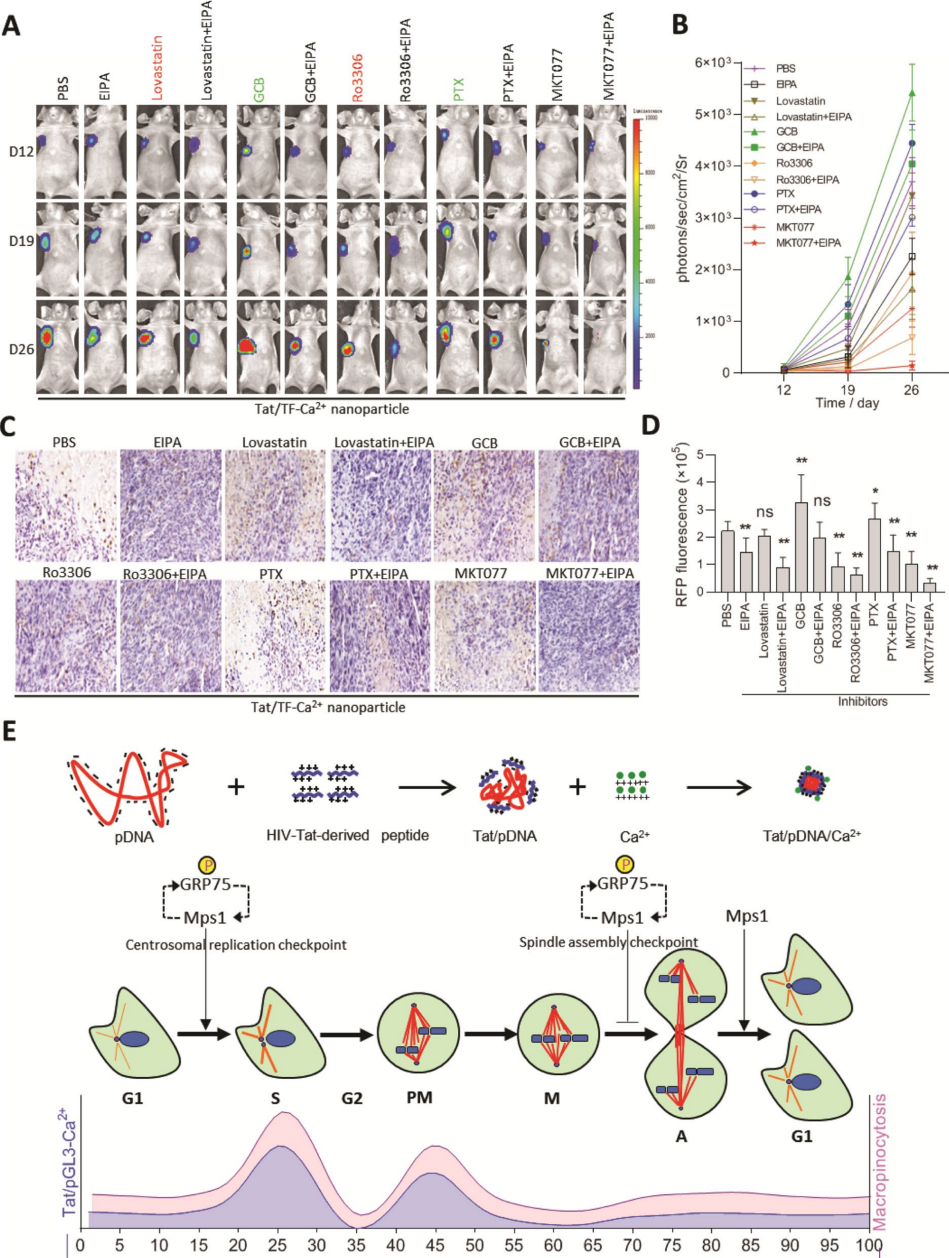
Bioluminescence imaging the delivery of Tat/TF-Ca^2+^ nanoparticles promoted by GRP75-driven, cell-cycle-dependent macropinocytosis. **(A)** In vivo analysis of the targeted delivery of Tat/TF-Ca^2+^ nanoparticles by Rluc imaging. Representative mice from different treatments are shown. **(B)** Quantitative analysis of mouse BLI signals being shown as photons/sec/cm2/sr. **(C**) Confirmation of Tat/TF-Ca^2+^ nanoparticles targeted delivery to ovarian cancer by anti-RFP immunohistochemistry, and **(D)** RFP-expressed signal from tumor tissue was quantified and analyzed. *P < 0.05, **P < 0.01. *ns* no difference. **(E**) Schematic illustration of GRP75-driven, cell-cycle-dependent macropinocytosis of Tat/pDNA-Ca^2+^ nanoparticles. Ca^2+^ addition markedly condensed Tat/pDNA nanocomplexes. Uptake of Tat/pDNA/Ca^2+^ nanoparticles was activated in cell-cycle S-phase and controlled by GRP75-driven, cell-cycle-dependent macropinocytosis. GRP75 interacts with Mps1, phosphorylates by Mps1, and forms a feedback-activation loop with MPS1. This signaling drives centrosome duplication, promotes cell-cycle S-phase enriched and M-phase arrested, and induces the macropinocytosis of Tat/TF-Ca^2+^ nanoparticles upregulated in these two sub-phases

To further determine the role of GRP75-driven, cell-cycle-dependent macropinocytosis on delivery of Tat/TF-Ca^2+^ nanoparticles, RLuc signal from TF plasmid was also investigated. As expected, Tat/TF-Ca^2+^ nanoparticles were specifically accumulated at OC site (Fig. 8A, B). When EIPA or MKT077 were further introduced, the OC-located RLuc signal from nanoparticles was sharply decreased, and was almost diminished after the co-injection of the two inhibitors. Weaker OC-targeted RLuc signal was observed after intratumoral co-injection with lovastatin or Ro3306, whereas further co-injection with GCB or PTX brought stronger RLuc signal at OC site (Fig. 8A, B). Again, when EIPA was introduced together with these cell-cycle inhibitors, synergistic TF RLuc signal was observed in lovastatin- or Ro3306-injected mice, and reversed TF RLuc signal were observed in GCB- or PTX-injected mice (Fig. 8A, B). The contrasted targeting of Tat/TF-Ca^2+^ nanoparticles to OC and distinct tumor-regression effect caused by different inhibitors was further confirmed by ex vivo RFP imaging and histological staining of RFP expression on resected OC tissues (Fig. 8C, D). Noticeably, no visceral toxicity was observed in OC-bearing mice treated by Tat/TF-Ca^2+^ nanoparticles with/without aforementioned inhibitors (Additional file 2: Fig. S13). Together, these results suggest that GRP75-driven, cell-cycle-dependent macropinocytosis dominantly underlies Tat/TF-Ca^2+^ nanoparticle-based suicide gene therapy in OC (Fig. 8E).

## Conclusions

In summary, Ca^2+^ addition tightly condensed Tat/pDNA complexes into small, nontoxic nanoparticles. Macropinocytic uptake of Tat/pDNA-Ca^2+^ nanoparticles was activated in S-phase and persisted in M-phase of cell-cycle. Over-expression or phosphorylation of GRP75 promoted MPS1-controled centrosome duplication and cell-cycle progress, but also drive cell-cycle-dependent macropinocytosis of Tat/pDNA-Ca^2+^ nanoparticles. Arresting cell-cycle at S-phase markedly enhanced the delivery efficiency of Tat/pDNA-Ca^2+^ nanoparticle, whereas inhibition of GRP75 reduced its macropinocytic uptake. Together, these data highlight that mitochondrial chaperone GRP75 moonlights as biphasic driver underlying cell-cycle-dependent macropinocytosis of Tat/pDNA-Ca^2+^ nanoparticles in ovarian cancer.

## Experimental section

### Cell culture, plasmids, antibodies, and reagents

Skov3, Cos7 and 293 T cells were obtained from the Type Culture Collection of the Chinese Academy of Sciences (China). Skov3 labeled with DF reporter gene were created by our lab [7]. GRP75 (wt)-, GRP75 (T62A/S65A)- and GRP75(T62D/S65D)-pEGFPC1 plasmids were as previously constructed [28, 61]. Lenti-CRISPR V2, psPAX2 and pCMV-VSVG plasmids (Addgene) were used to produce lentivirus and establish GRP75-knock-down (KD) cell lines [27]. pLV-EF1a-MCS-IRES-GFP-Puro, pGag-Pol, pRev and pVSV-G plasmids (Biosettia) were used to produce lentivius and create GRP75-overexpression (OE) cell lines [24]. pGL3 luciferase reporter plasmid (Promega) was used for monitoring in vitro transfection efficiency [6]. Lentiviral double fusion (DF) plasmid, carrying a ubiquitin promoter driving firefly luciferase and enhanced green fluorescence protein reporter gene (Fluc-eGFP), and triple fusion (TF) plasmid, carrying monomeric red fluorescence protein, renilla luciferase and herpes simplex virus truncated thymidine kinase reporter gene (RFP-Rluc-HSV-ttk) were used for targeted imaging and gene therapy [7].

Mouse anti-GRP75 antibody (Ab) (sc-133137) and anti-VDAC1 (sc390996), rabbit anti-B-actin Ab (sc-7210) were obtained from Santa Cruz. Rabbit anti-Arf6 Ab (381658) was obtained from Zenbio. Mouse anti-RFP Ab (31101ES60) was obtained from Yeasen. Mouse anti-β-actin Ab (66009), anti-r-tubulin Ab (66320), and rabbit anti-GRP75 Ab (14887), anti-r-tubulin Ab (15176), anti-MPS1 Ab (10381), and goat anti-mouse Ab-AF488 (A-11001), anti-mouse Ab-AF647 (SA00006-1), anti-mouse Ab-AF594 (SA00006-3), anti-rabbit Ab-AF488 (SA00006-3) were obtained from Proteintech. HIV-Tat-derived peptide (GRKKRRQRRRPPQC) was obtained from GL Biochem Ltd. DiYO-1 (17580) and DiYO-3 (17581) were obtained from AATbio. Luciferase Reporter Gene Assay Kit (11401ES60), Coelenterazine H (40906ES03), D-Luciferin (40901ES02), TUNEL apoptosis Kit (40306ES), cell apoptosis kit (40306ES) and polybrene (40804ES) were obtained from Yeasen. Annexin V-FITC apoptosis detection kit (C1062L) was obtained from Beyotime. GCV, (HY-13637), MKT077 (FJ-776), RO3306 (HY-12529) and EIPA hydrochloride (HY-101840A) were obtained from MCE. SP-9000 SPlink Detection Kit (SP-9000) was obtained from ZSGB-BIO. Lovastatin (SL8280), GCB (G8970). PTX (SP8020), Sucrose (S8271) and HU (H8420) were obtained from Solarbio. 70 kDa Dextran-RD (D1841), BCA protein assay Kit (23225), Hochest33342 (B2261), propidium Iodide (P4170), puromycin (A1113803), lipofectamine 3000 (L3000015) and other chemicals were obtained from Thermo Fisher.

### Cell synchronization and cell-cycle analysis

Synchronizing cells at different sub-phases of the cell-cycle were performed by the previous report with some modifications [24, 62] (Fig. 2E). Briefly, cells were treated with HMG-CoA reductase inhibitor lovastatin (20 uM) for G1-phase arrested, or treated with low dosage of GCB (10 nM) for S-phase blocked [63], or treated with CDK1 inhibitor RO3306 (10 uM) for G2-phase arrested, or treated with PTX (0.1 mM) for M-phase arrested [64]. To assess the phase-distribution of cell-cycle, treated cells were collected, fixed in 70% ethanol overnight, incubated with RNase A (4 °C, 30 min), and then stained with propidium iodide (50 μg/ml) and evaluated by FACS Calibur (BD). Cell-cycle distribution of GRP75-KD or -OE cells was similarly analyzed as describing above.

### Gene stable knock-down and over-expression

CRISPR/Cas9 lentivirus system was used to produce stable GRP75-KD cell lines. Three independent GRP75 targeting sgRNA were generated as previously described [24, 27], and the most efficient targeting shRNA (5’-CACCGTGGAATGCCGGCCAAGCGAC-3’, 5’-AAACGTCGCTTGGCCGGCATTCCAC-3’) was adopted for creating stable cell lines. cDNA sequence of wild-type GRP75 (wt), unphospho-type GRP75 (T62A/S65A) and phosphoimic-type GRP75 (T62D/S65D) was respectively cloned into pLV-EF1a-MCS-IRES-GFP-Puro lentiviral gene expression vector for producing stable GRP75-OE cell lines (Additional file 2: Fig. S8). Efficiency of GRP75-KD or OE was checked by Western blotting.

### Preparation and characterization of nanoparticles

Tat/pDNA-Ca^2+^ nanoparticles were similarly prepared as previously described [11, 12]. Briefly, pDNA (pGL3 or TF plasmid) were respectively labeled with DIYO-1 or DIYO-3 at a 1:5 ratio of the dye molecule to DNA base pair (dye/bp). Tat/pDNA complexes were formulated at charge ratios (positive charges of peptide to negative charges of pDNA, N/P) of 1, 5, 10 and 20 by drop-wise addition of Tat peptide to pDNA. These mixtures were prepared at a constant pDNA concentration (100ug/mL), while the N/P ratios of the complexes were varied. The mixture was vortexed for 20 min, and aqueous Tat/pDNA mixtures with desirable N/P ratio were gently mixed with CaCI_2_ solutions (113 mM). Resulted mixtures were equilibrated at 4 °C, centrifuged, washed with PBS, and the pellet contained the target nanoparticles.

Morphology of Tat/pDNA-Ca^2+^ nanoparticles was negatively stained with 2% uranyl acetate dihydrate and checked under the transmission electron microscopy (Philips Tecnai G2 20 S-TWIN). Mean particle size and the zeta potential of the nanoparticles were determined by DLS (ZETAPALS/BI-200SM, BROOKHAVEN). Stability of the nanoparticles was tested by electrophoretic retardation and DNase I protection assays. All these characterizations were similarly performed as previously described [6, 7].

### Luciferase gene expression and fluoresce quantification

Tat/pDNA-Ca^2+^ nanoparticles (10 ug pDNA) diluted in 2 mL incomplete medium with/without cell-cycle inhibitors (Fig. 2E) were added to parallel wells (cell density: 2 × 10^4^ per cm^2^) at 37 °C for 4–14 h transfection. Before changing to complete medium, one aliquot of cells was thoroughly washed by PBS/0.5 M NaCl, lysed and determined the fluoresce intensity by the fluorescence microplate reader (FLUOstar Omega). The RFU (relative fluoresce units) was normalized for protein content. The other aliquot of cells were changed with complete medium for an additional 44 h growth, and cells were lysed, transferred for the luciferase expression analysis. The light emission was determined with signal integration over 10 s at 25 °C. The activity (relative light units, RLU) was normalized for protein content.

### Uptake and co-localization analysis of nanoparticles

Cellular uptake of Tat/pDNA-Ca^2+^ nanoparticles was measured by confocal imaging analysis as previously described [6, 7]. Cells in chamber slides were first washed with serum-free medium, then incubated with fluorescent labeled particles/nanoparticles. Incubation conditions for endocytosis marker (Dextran-RD) and DiYO1/3-labeled nanoparticles were optimized first. Cells were incubated with DiYO-1/3-labeled nanoparticles at 37 °C, quick-rinsed with PBS/0.5 M NaCl, nuclei-stained with Hochest33342, and immediately image-acquired using a confocal microscope (Olympus FV 1000). The images were processed by ImageJ software. For the uptake determined by flow cytometry, trypan blue was added to quench cell surface-associated fluorescence before analysis.

To compare the uptake in synchronized culture to that in non-synchronized one, care was taken to ensure that a similar number of cells were exposed to nanoparticles. In addition, prior to addition of fluorescent particles, various cell-cycle arrested conditions were achieved via 24 h pre-incubation with serum-free media containing lovastatin, GCB, RO3306, and PTX. These drugs were made in DMSO, diluted in PBS, and the final concentration of DMSO was less than 0.4%.

### Centrosome re-duplication and immunofluorescence

Centrosome re-duplication assay was performed as previously described [54]. Briefly, GRP75-KD or -OE cells were grown for 24 h, and then started for treating with or without HU for 60 h. The number of centrosomes per cell and the co-localization of GRP75 and MPS1 were determined by co-immunostaining with anti-GRP75 (1:200), anti-MPS1 (1:100) and anti-γ-tubulin (1:100) antibodies. Target protein co-localizing at centrosome was detected with either Alexa 488- or Alexa 594-conjugated goat secondary antibody and examined under a confocal microscope using a 60 × objective lens. Images were analyzed with Image J software, and co-localization was calculated as the Rank Weighted Coefficient (RWC) [65].

### Centrosome isolation and western blotting

Centrosome from asynchronously and synchronously growing cells was isolated by sucrose density-gradient centrifugation method as previously described [54, 66]. Protein concentration of lysate samples in RIPA buffer was measured using the BCA assay, resolved by SDS-PAGE, blotted onto PVDF membranes, and then analyzed with different Abs diluted as follows: anti-GRP75 Ab (1:1000), anti-r-tubulin (1:2000), anti-MPS1 Ab (1:300). anti-Arf6 (1:1000), anti-VDAC1 Ab (1:1000), anti-B-actin Ab (1:2000).

### Mice model and nanoparticle and drug treatments

NOD/SCID female mice (6–8 week-old, from Beijing HFK Bioscience) were randomized into 12 groups (6 mice per group), and injected subcutaneously with 2 × 105 DF-labeled Skov3 cells into the right armpit. OC-bearing mice were firstly treated with Tat/pDNA-Ca2 + nanoparticles (1 mg/kg) by tail vein injection every week, then followed by intratumoral injections of EIPA (10 mg/kg) or MKT077 (7.5 mg/kg). Combined intratumoral injections of cell-cycle inhibitors, lovastatin (12.5 mg/kg), GCB (35 mg/Kg), Ro3306 (4 mg/kg), PTX (10 mg/kg), were parallel performed once a week in the additional nanoparticle groups. All mice were administered intraperitoneally with GCV (50 mg/kg) from day 11 till the end of experiment (Fig. 7A, B).

### Bioluminescence imaging of ovarian cancer in vivo

The fate of transplanted Skov3(DF) cells and the distribution of the aforementioned nanoparticles in living mice was tracked by bioluminescence imaging (Fig. 7B) as previously described [7]. Briefly, mice were detected bioluminescence intensity to assess for Rluc or Fluc expression using the In Vivo Imaging System (IVIS 200, XENOGEN). Following anesthesia and placing on the heating plate, mice received tail-vein-injection with coelenterazine IV (2.5 mg/kg) were imaged immediately afterwards for 4 min to assess Rluc expression (signal from nanoparticles). One hour later, mice injected intraperitoneally with D-Luciferin (150 mg/kg) were performed 1 s to 5 min scans to assess Fluc expression. Bioluminescence signals were quantified by Living Image 4.3 software, and presented as units of average photons per second per centimeter square per steridian (photons/s/cm2).

### Histology and immunohistochemistry

Tumor and other organ tissues from sliced mice were isolated, fixed, and processed for tissue paraffin sections as previously described [7]. To determine the TF plasmid expression, tissue slices were stained with anti-RFP Ab (1:200) and DAPI (2ug/mL) for nuclear counterstaining based on SPlink Detection Kit. Hematoxylin and eosin (HE) staining were performed on tissue sections which observed under the optical microscope (Olympus).

### Cell death and tissue apoptosis assays

Cell death was assessed by using annexin V-PI apoptosis detection kit followed by flow cytometry analysis. Data was recorded from 1 × 10^4^ cells in triplicate per condition. Tumor tissues were fixed for 24 h in 4% paraformaldehyde, and then processed to cryosections. In situ fluorescent TUNEL staining was done by using TUNEL apoptosis detection kit. Images were obtained by fluorescent microscopy (Olympus) and quantified with ImageJ software (NIH).

### Bioinformatics analysis

Cyclebase 3.0 [67], a multi-organism database including mRNA and protein expression information on cell-cycle regulation and phenotypes was used to predict the expression level of GRP75 in sub-phases of the cell-cycle as previously described [24].

### Statistical analyses

All data were obtained from three independent experiments, and are presented as mean ± SD. For two-sample comparisons against control, unpaired Student’s t-test was used. One-way analysis of variance with Dunn’s multiple comparison was used to evaluate the statistical significance among multiple groups. Graphs were created using GraphPad Prism 8.0 software.

## Supplementary Information

**Additional file 1. Table S1.** Mean size and zeta potential of Tat/pDNA-Ca^2+^ nanoparticles.

**Additional file 2: Fig. S1.** Granularity and electric potential analysis of Tat/pGL3 and Tat/pGL3-Ca^2+^ particles. **Fig. S2.** High-concentration, long-term treatment with Tat/pGL3-Ca^2+^ nanoparticles triggers necrotic apoptosis. **Fig. S3.** Stability characteristics of Tat/pDNA-Ca^2+^ nanoparticles in culture media or mice serum. **Fig. S4.** Unpackaging of Tat/pDNA complexes or Tat/pDNA-Ca^2+^ nanoparticles by heparin displacement of pDNA. **Fig. S5.** Tat/pGL3-Ca2+ nanoparticles mainly use macropinocytosis for uptake. **Fig. S6.** EIPA treatment inhibited the uptake and expression of Tat/pGL3-Ca^2+^ nanoparticles. **Fig. S7.** Tat/pDNA-Ca^2+^ nanoparticles do not interfere with sub-phase distribution of cell-cycle. **Fig. S8.** Construction of recombinant lentiviral plasmids for GRP75 over-expression (**A)** and knock-down (**B**). **Fig. S9.** Highly expression or phosphorylated activation of GRP75 promotes centrosome duplication in Cos7 cells, and GRP75 mainly localizes in duplicated centrosome. **Fig. S10.** Highly expression or phosphorylated activation of GRP75 promotes itself and Mps1 co-translocating to centrosome in Cos7 cells. **Fig. S11.** GRP75 and Mps1 co-localized with r-tubulin only in duplicating centrosome. **Fig. S12.** Quantification of apoptotic cells in ovarian tumor with different treatments. **Fig. S13.** H&E staining of hearts, livers, spleens, lungs, kidneys and tumor tissues from mice with different treatments.

## Abbreviations

Ab: Antibody
CaCI_2_: Calcium chloride
DLS: Dynamic light scattering
DF: Double fusion
EIPA: 5-(N-ethyl-N-isopropyi)-amiloride
G1-phase: Gap phase 1
G2-phase: Gap phase 2
GCB: Gemcitabine
GRP75: 75-kDa glucose-regulated protein
HU: Hydroxyurea
IHC: Immunohistochemistry
I-phasse: Interphase
KD: Knock-down
M-phase: Mitosis phase
Mps1: Monopolar spindle kinase 1
N/P: Positive charges of peptide to negative charges of pDNA
OE: Over-expression
pDNA: Plasmid DNA
PTX: Paclitaxel
RD: Rhodamine
RLU: Relative light unit
RFU: Relative fluorescence unit
S-phase: DNA synthesis phase
Tat: HIV-Tat-derived peptide
TEM: Transmission electron microscope
TF: Triple fusion
