# Supplementary material for: GRP75-driven, cell-cycle-dependent macropinocytosis of Tat/pDNA-Ca2+ nanoparticles underlies distinct gene therapy effect in ovarian cancer
Source: J Nanobiotechnology. 2022 Jul 20;20:340. doi: 10.1186/s12951-022-01530-6 (PMC9301890; doi:10.1186/s12951-022-01530-6)
Supplement: Supplementary file 1 — Additional file 1. Table S1. Mean size and zeta potential of Tat/pDNA-Ca2+ nanoparticles. [file 12951_2022_1530_MOESM1_ESM.doc]

**Table S1 Mean size and zeta potential of Tat/pDNA-Ca^2+^ nanoparticles**

| Tat/pGL3 | | N/P=1 | N/P=5 | N/P=10 | N/P=20 |
| --- | --- | --- | --- | --- | --- |
| PBS | Z-average (d.nm) | 1160 | 780 | 520 | 510 |
|  | Z-potential (mV) | -47.2 | 3.4 | 3.2 | 5.1 |
|  | PDI | 0.78 | 0.56 | 0.42 | 0.40 |
| CaCI_2_ | Z-average (d.nm) | 820 | 620 | 382 | 218 |
|  | Z-potential (mV) | -43.0 | 2.6 | 3.8 | 4.8 |
|  | PDI | 0.69 | 0.46 | 0.23 | 0.20 |

**Notes**: N/P ratio is the molar ratio of total free amino groups (positive charge) in Tat peptide to total free phosphate groups (negative charge) in pDNA taken in solution. The detailed calculation was described in ‘Biomater Res. 2016, 20(28):1-8’ and ‘Vaccine. 2002,20(17-18):2303-17. The particle sizes shown here are hydrodynamic diameters.
