# Supplementary material for: GRP75-driven, cell-cycle-dependent macropinocytosis of Tat/pDNA-Ca2+ nanoparticles underlies distinct gene therapy effect in ovarian cancer
Source: J Nanobiotechnology. 2022 Jul 20;20:340. doi: 10.1186/s12951-022-01530-6 (PMC9301890; doi:10.1186/s12951-022-01530-6)
Supplement: Supplementary file 2 — Additional file 2: Fig. S1. Granularity and electric potential analysis of Tat/pGL3 and Tat/pGL3-Ca2+ particles. Fig. S2. High-concentration, long-term treatment with Tat/pGL3-Ca2+ nanoparticles triggers necrotic apoptosis. Fig. S3. Stability characteristics of Tat/pDNA-Ca2+ nanoparticles in culture media or mice serum. Fig. S4. Unpackaging of Tat/pDNA complexes or Tat/pDNA-Ca2+ nanoparticles by heparin displacement of pDNA. Fig. S5. Tat/pGL3-Ca2+ nanoparticles mainly use macropinocytosis for uptake. Fig. S6. EIPA treatment inhibited the uptake and expression of Tat/pGL3-Ca2+ nanoparticles. Fig. S7. Tat/pDNA-Ca2+ nanoparticles do not interfere with sub-phase distribution of cell-cycle. Fig. S8. Construction of recombinant lentiviral plasmids for GRP75 over-expression (A) and knock-down (B). Fig. S9. Highly expression or phosphorylated activation of GRP75 promotes centrosome duplication in Cos7 cells, and GRP75 mainly localizes in duplicated centrosome. Fig. S10. Highly expression or phosphorylated activation of GRP75 promotes itself and Mps1 co-translocating to centrosome in Cos7 cells. Fig. S11. GRP75 and Mps1 co-localized with r-tubulin only in duplicating centrosome. Fig. S12. Quantification of apoptotic cells in ovarian tumor with different treatments. Fig. S13. H&E staining of hearts, livers, spleens, lungs, kidneys and tumor tissues from mice with different treatments. [file 12951_2022_1530_MOESM2_ESM.doc]

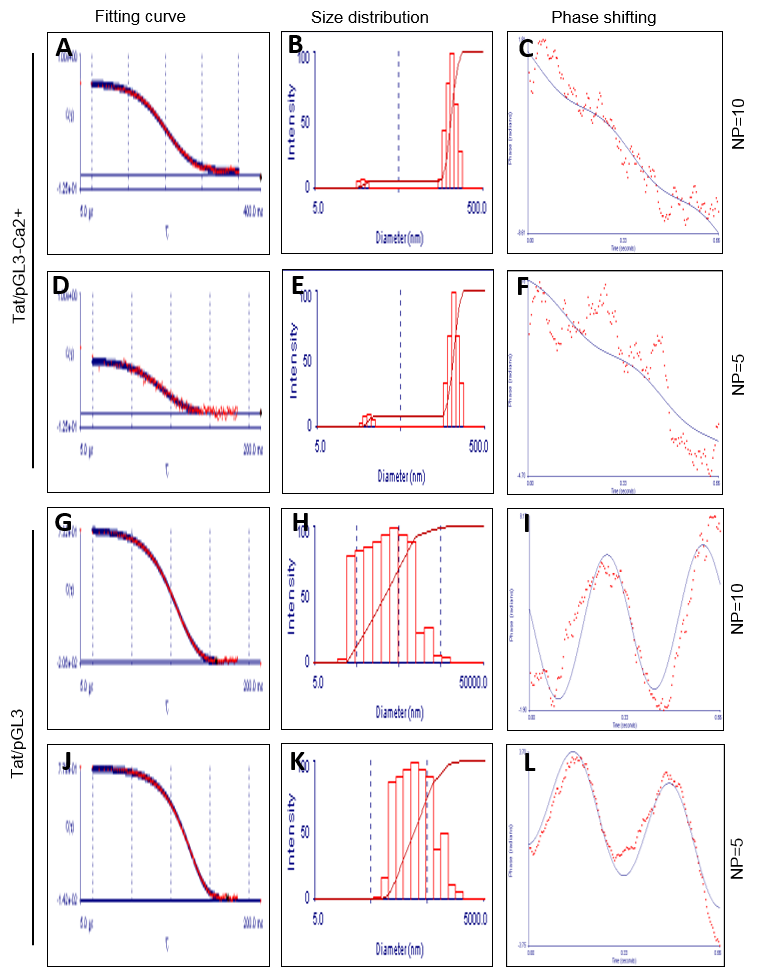


**Fig. S1 Granularity and electric potential analysis of Tat/pGL3 and Tat/pGL3-Ca^2+^ particles.** Representative results of fitting curve (A, D, G, I), size distribution (B, E, H, K), phase shifting (C, F, I, L) for Tat/pGL3 (A-F) and Tat/pGL3-Ca2+ (G-L) produced at different NP ratios.


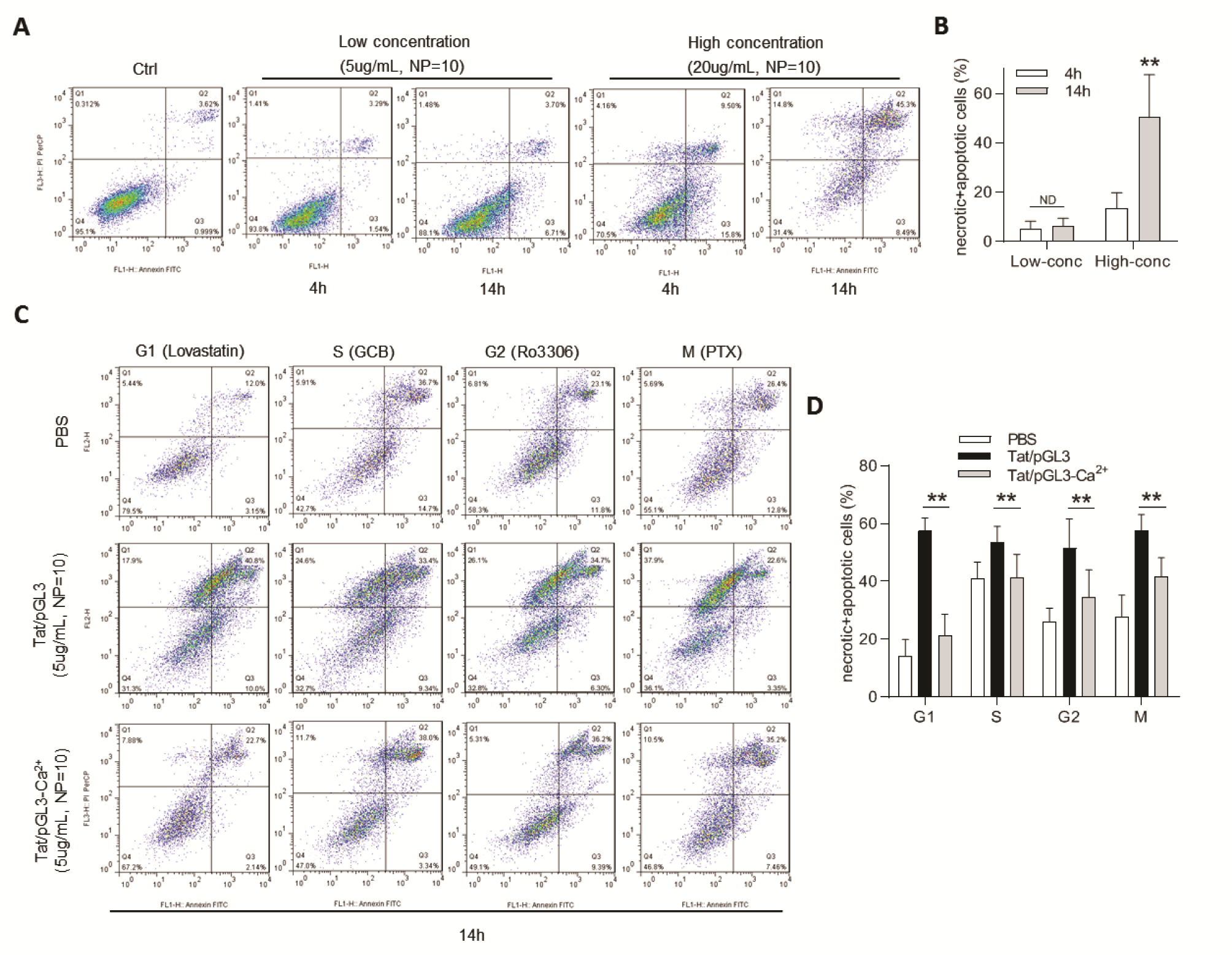


**Fig. S2 High-concentration, long-term treatment with Tat/pGL3-Ca^2+^ nanoparticles triggers necrotic apoptosis.** (**A**, **C**) Flow cytometry-based detection of apoptosis status in Skov3 cells: (**A**) incubating with low (5ug/mL), high (20ug/mL) concentration of Tat/pGL3-Ca^2+^ nanoparticles, (**C**) treating with sub-phase-arrested drugs and further incubating with Tat/pGL3 or Tat/pGL3-Ca^2+^ nanoparticles (5ug/mL, NP=10). (**B**, **D**) Apoptosis level in Skov3 cells was calculated for different treatments. n=3. Statistically significant differences in relation to short-time or no-particle treatments are shown: ***P* <0.01, **P* <0.05. Note: Different forms of particles were prepared and compared at constant N/P ratio, while the incubation time to cells or the final concentration of pDNA complexes were varied (A-B) or not varied (C-D) in these assays.


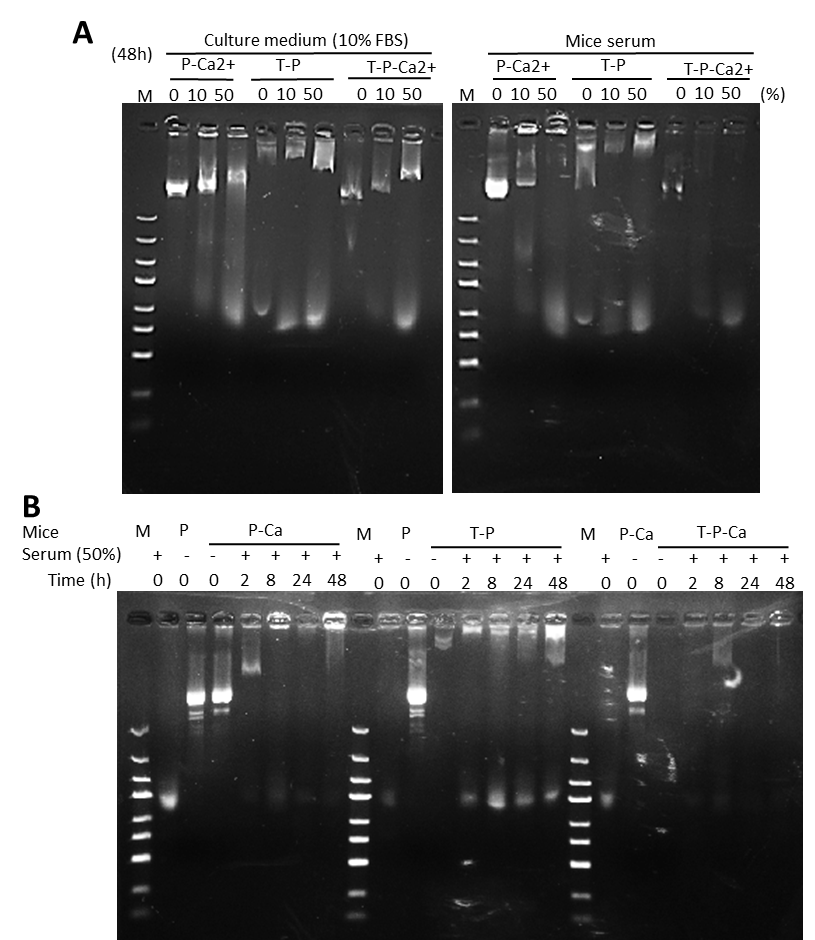


**Fig. S3** **Stability characteristics of Tat/pDNA-Ca^2+^ nanoparticles in culture media or mice serum**. (**A**) Agarose electrophoresis analysis of the stability of Tat/TF-Ca^2+^ nanoparticles incubated with cell culture medium (left panel) and mice serum (righlt panel), respectively. P-Ca^2+^: TF plasmid mixed with calcium chloride (113mM); T-P: TF plasmid compacted with Tat peptide (N/P=10); T-P-Ca^2+^: TF plasmid compacted with Tat peptide and 113mM calcium chloride (N/P=10). (**B**) Time-course checking the serum-stability of P-Ca^2+^, T-P and T-P-Ca^2+^ nanoparticles by agarose electrophoresis analysis. N/P ratios=10. (-) no serum was mixed. M: DNA ladder.


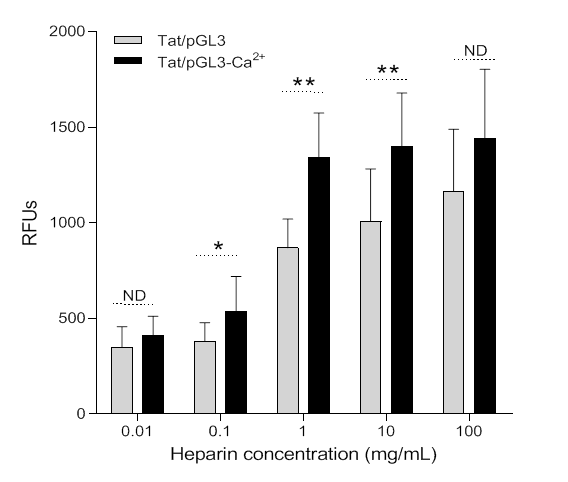


**Fig. S4 Unpackaging of Tat/pDNA complexes or Tat/pDNA-Ca^2+^ nanoparticles by heparin displacement of pDNA**. Results are presented as mean±SD (n=3).


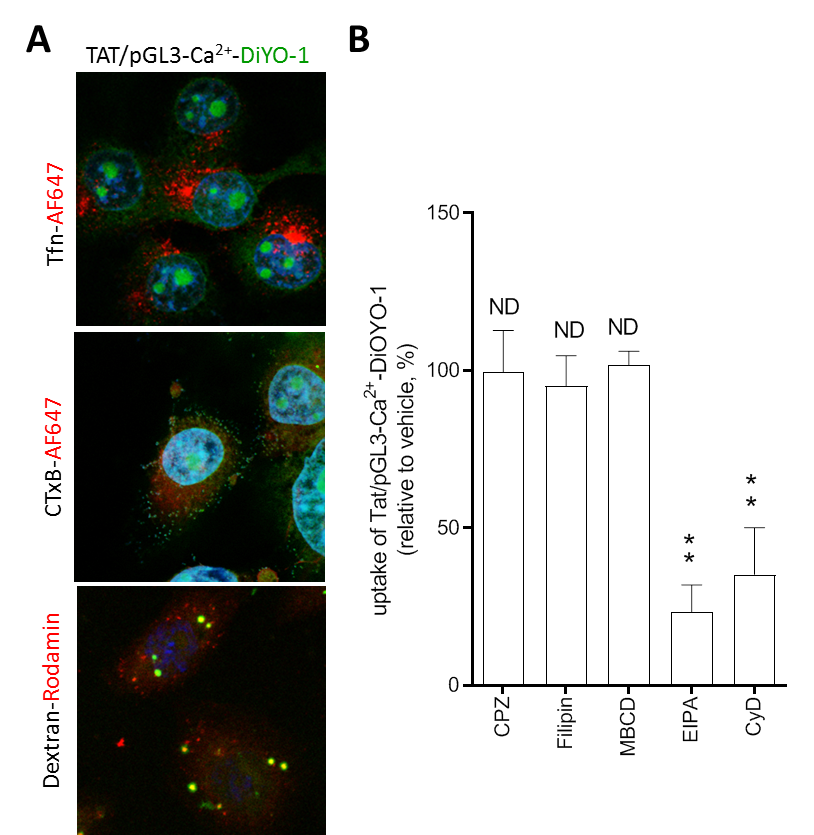


**Fig. S5 Tat/pGL3-Ca2+ nanoparticles mainly use macropinocytosis for uptake.** (A) Confocal imaging of the co-localization of Tat/pGL3-Ca^2+^-DiYO-1 nanoparticles (N/P=10) with different markers of endocytosis pathway. Dextran-RD (25ug/mL, red emission signal) , CTxB-AF647 (10 μg/mL, red emission signal), Tfn-AF647 (25 μg/mL, red emission signal). After co-incubation for 1 hour, SKOV3 cell nuclei were stained with Hoest3312 (blue emission signal). (B) The uptake levels of Tat/pGL3-Ca^2+^-DiYO-1 nanoparticles (N/P=10) in SKOV3 cells were quantified and analyzed by flow cytometry. Cells were respectively pretreated with endocytosis inhibitors: Cytochalasin D (CyD, 5uM), 5-(N-Ethyl-N-isopropyi)-amiloride (EIPA, 10uM), Methyl-b-cyclodextrin (MBCD, 1mM), Filipin (5ug/mL), and Chlorpromazine (CPZ). About 10,000 cells were counted per treatment. Statistically significant differences compared with dimethyl sulfoxide (DMSO) (,0.4%, final concentration) treated cells (Ctrl) are shown, ** *P*<0.01.


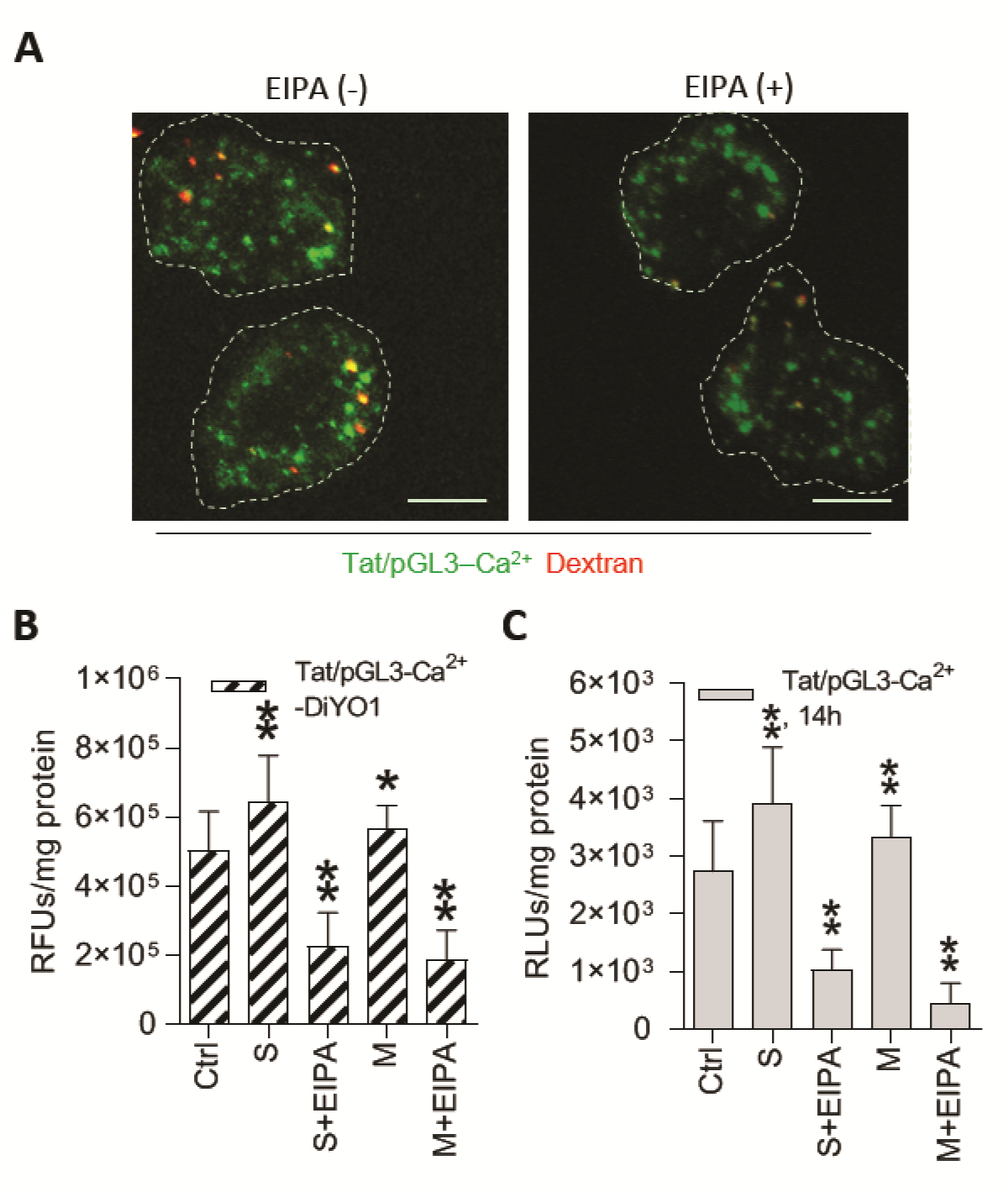


**Fig. S6 EIPA treatment inhibited the uptake and expression of Tat/pGL3-Ca^2+^ nanoparticles.** (**A**) Confocal imaging the uptake of Tat/pGL3-Ca^2+^-DiYO1 nanoparticles together with Dextran-RD in Skov3 cells with/without EIPA pre-incubation. (**B**, **C**) Internalized fluorescence and Luciferase activity of Tat/pGL3-Ca^2+^ nanoparticles (14 hours transfection) in sub-phase-arrested Skov3 cells treated with/without EIPA. n=3. Statistically significant differences in relation to Tat/pGL3 treatment are shown: ***P* <0.01, **P* <0.05.


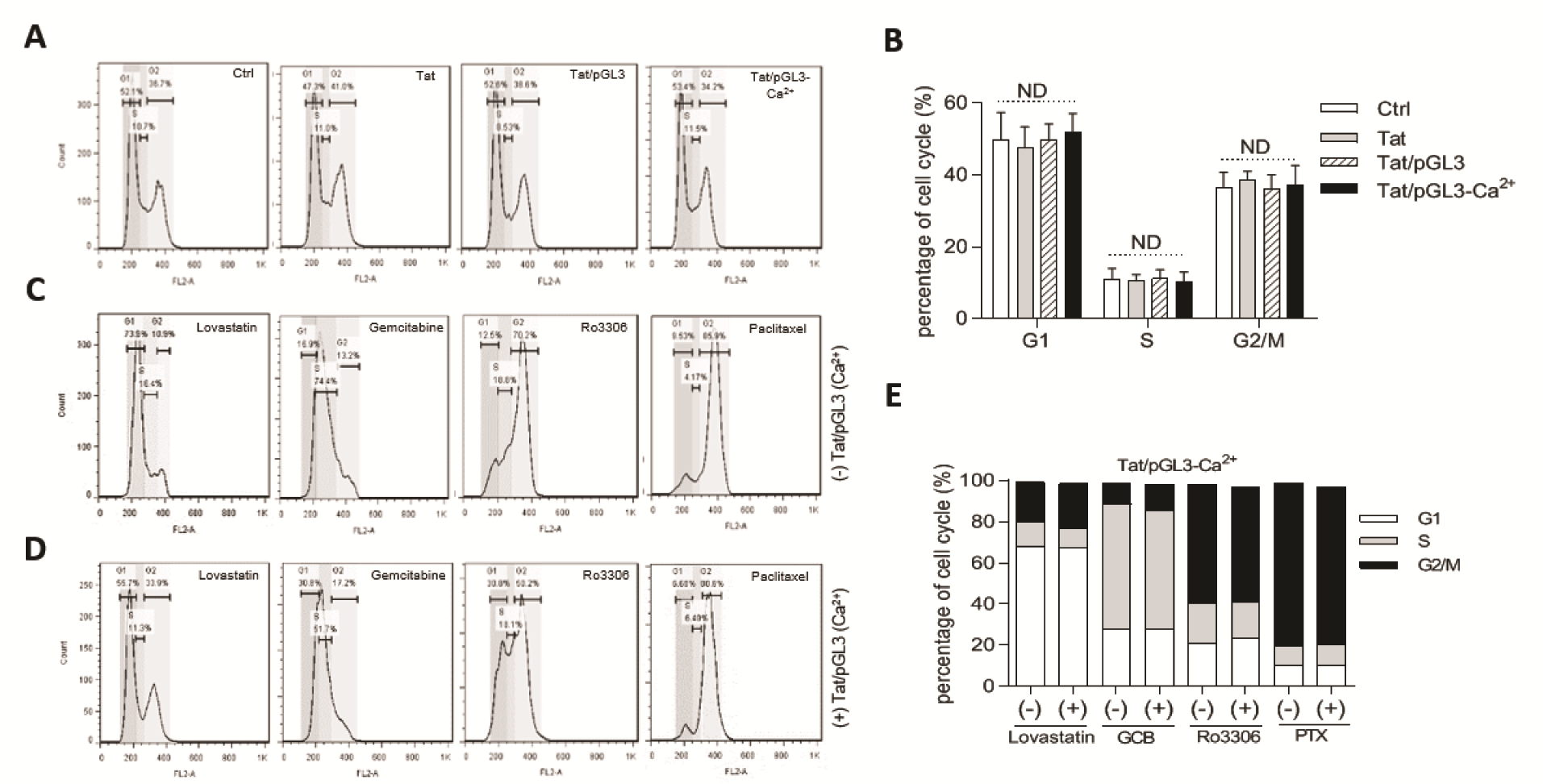


**Fig. S7 Tat/pDNA-Ca^2+^ nanoparticles do not interfere with sub-phase distribution of cell-cycle.** Cell-cycle was determined by propidium iodide staining and flow cytometry analysis in Skov3 cells: (**A**) Incubating with Tat, Tat/pGL3 and Tat/pGL3-Ca^2+^ nanoparticles, (**C**) treating with sub-phase-arrested drugs for cell-cycle enrichment. Atorvastatin, G1-phase arrest; Gemcitabine, S-phase arrest; RO3306, G2-phase arrest; Paclitaxel, M-phase arrest, (**D**) treating with sub-phase-arrested drugs and further incubating with Tat/pGL3-Ca^2+^ nanoparticles for transfection. (**B**, **E**) Sub-phase distribution of cell-cycle was calculated and summarized for different treatments. n=3. Statistically significant differences in relation to Ctrl (no drug treatment) are shown: ND, no difference.


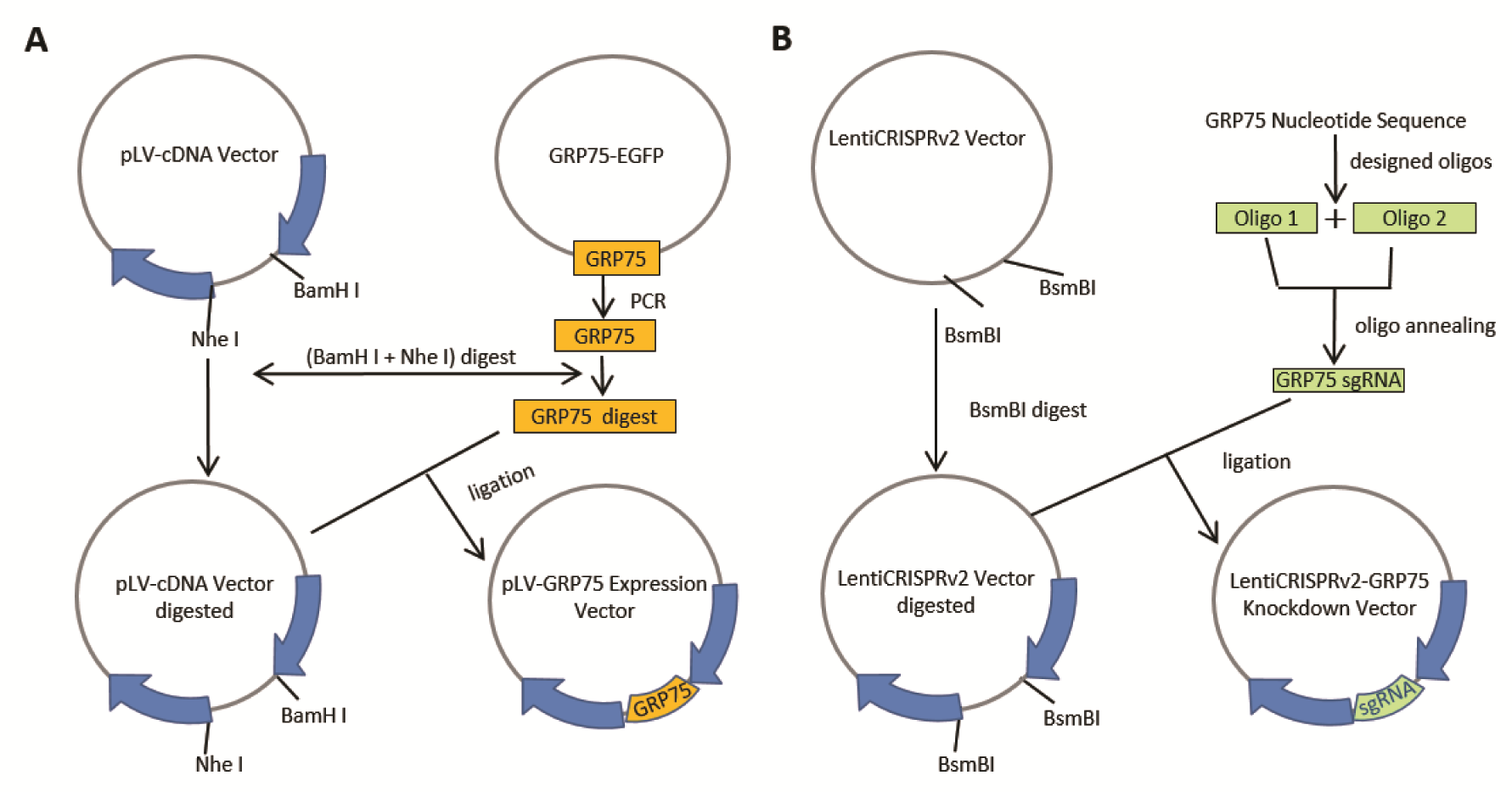


**Fig. S8 Construction of recombinant lentiviral plasmids for GRP75 over-expression (A) and knock-down (B).**


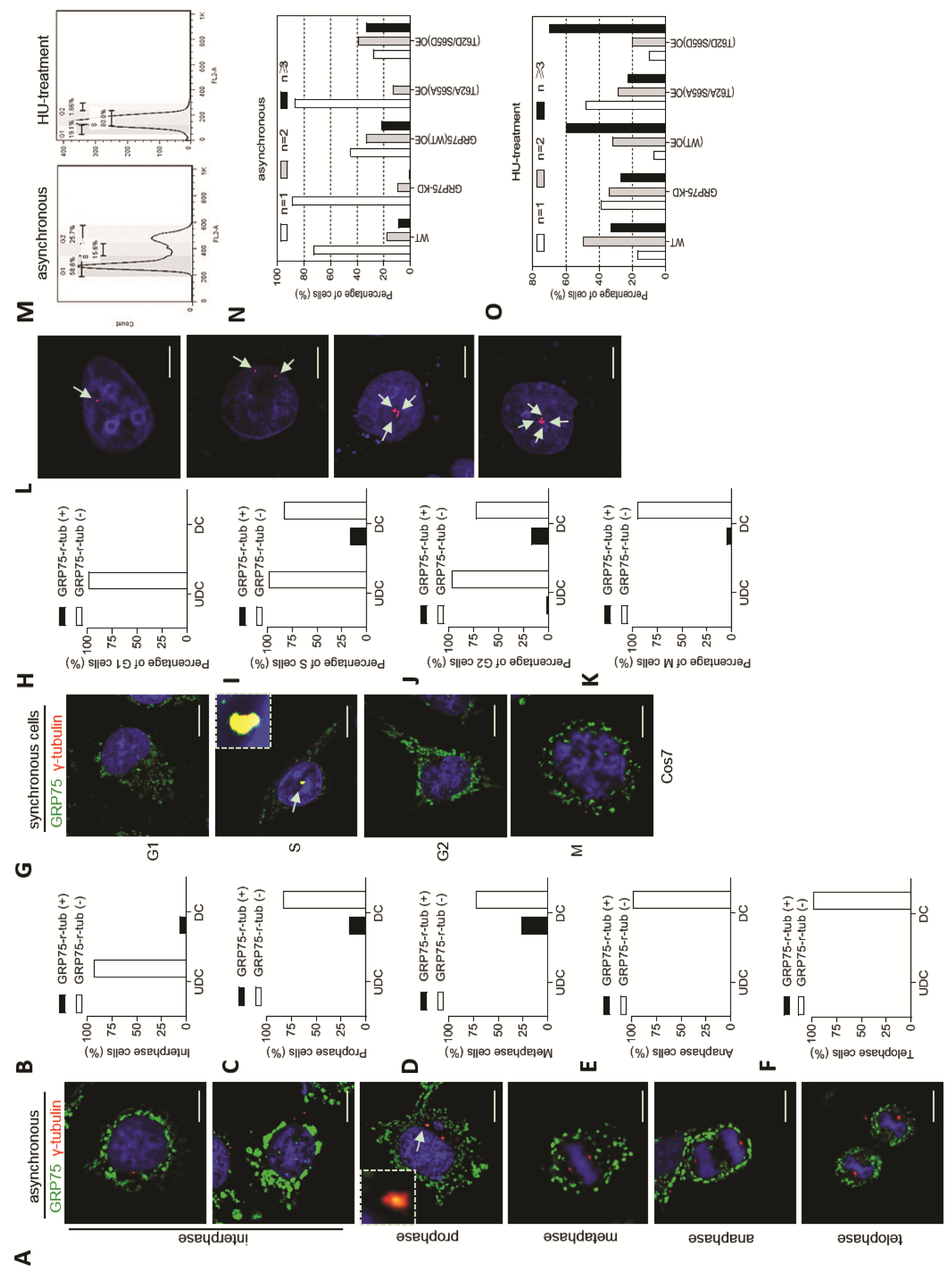


**Fig. S9 Highly expression or phosphorylated activation of GRP75 promotes centrosome duplication in Cos7 cells, and GRP75 mainly localizes in duplicated centrosome.** (**A**, **G**) Confocal imaging the co-localization of GRP75 with γ-tubulin in asynchronously and synchronously growing Cos7 cells. Arrows point to the positions of centrosomes. Images placed on the up-right showing the magnification of areas indicated by arrows. Scale bar, 10 μm. (**B**-**F**, **H**-**K**) The frequencies of GRP75 co-localization with γ-tubulin among unduplicated and duplicated centrosomes were determined and plotted. (**L**) Confocal imaging the replicated and non-replicated centrosomes in hydroxyurea (HU)-exposed cells, and (**M**) cell-cycle was determined by flow cytometry analysis. (**N**, **O**) The frequency of centrosomes per cell was scored and shown in asynchronously and synchronously growing Cos7 cells with GRP75-KD or -OE.


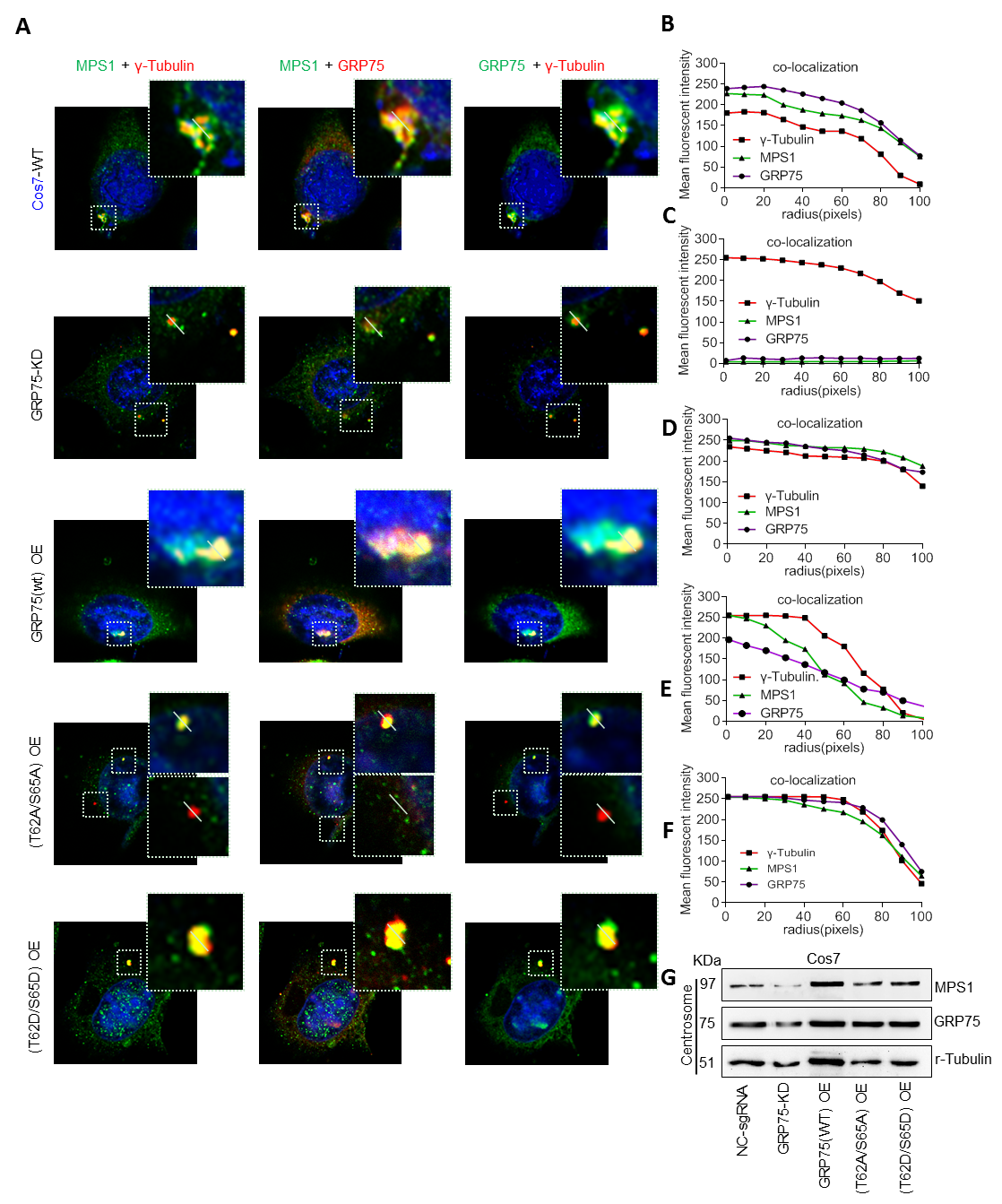


**Fig. S10 Highly expression or phosphorylated activation of GRP75 promotes itself and Mps1 co-translocating to centrosome in Cos7 cells.** (**A**) Confocal imaging the co-localization of MPS1 with γ-tubulin, GRP75 with γ-tubulin, GRP75 with γ-tubulin in Cos7 cells with GRP75-KD or -OE. (**B**-**F**) Fluorescent intensities along the central line across centrosome were plotted, and staining signal distribution curves of GRP75, MPS1 and γ-tubulin were determined by ImageJ software and are shown. (**G**) Western blot determined the centrosomal translocation level of GRP75 and MPS1 in Cos7 cells with GRP75-KD or -OE.


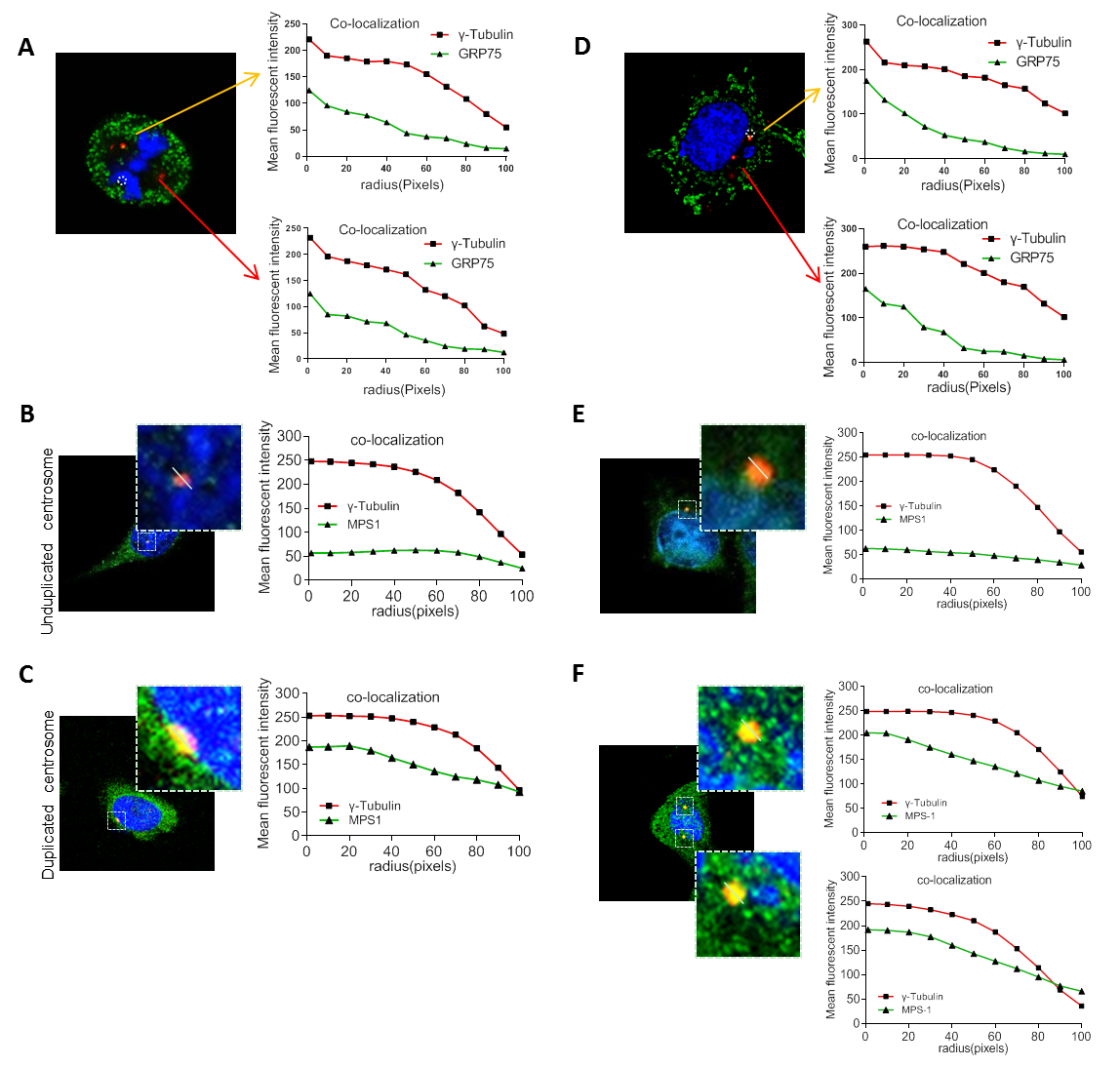


**Fig. S11 GRP75 and Mps1 co-localized with r-tubulin only in duplicating centrosome.** Confocal imaging the co-localization of GRP75 and γ-tubulin (**A**, **D**), MPs1 with γ-tubulin (**B**, **C**, **E**, **F**) in asynchronously growing Skov3 (**A,** **B**, **C**) and Cos7 (**D**, **E**, **F**) cells. Fluorescent intensities along the central line across centrosome were plotted, and staining signal distribution curves of GRP75, MPS1 and γ-tubulin were determined by ImageJ software and are shown.


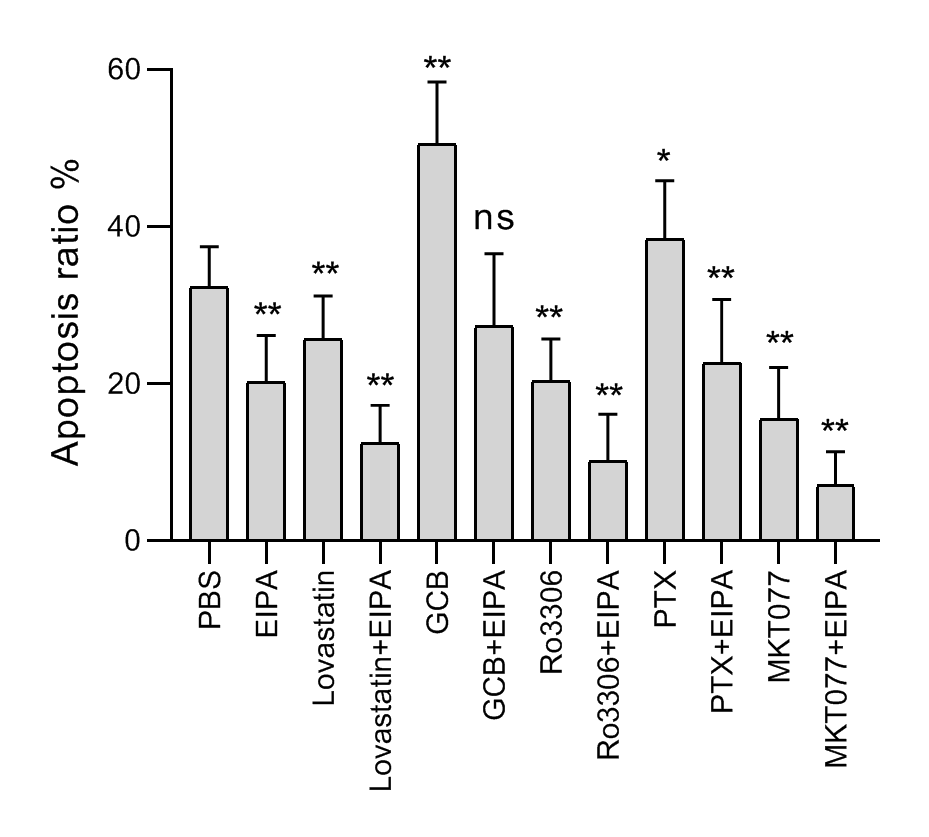


**Fig. S12** **Quantification of apoptotic cells in ovarian tumor with different treatments.** TUNEL-assay was used to determine the apoptosis, and the ratio of apoptosis was quantified. **P* < 0.05, ***P* < 0.01.


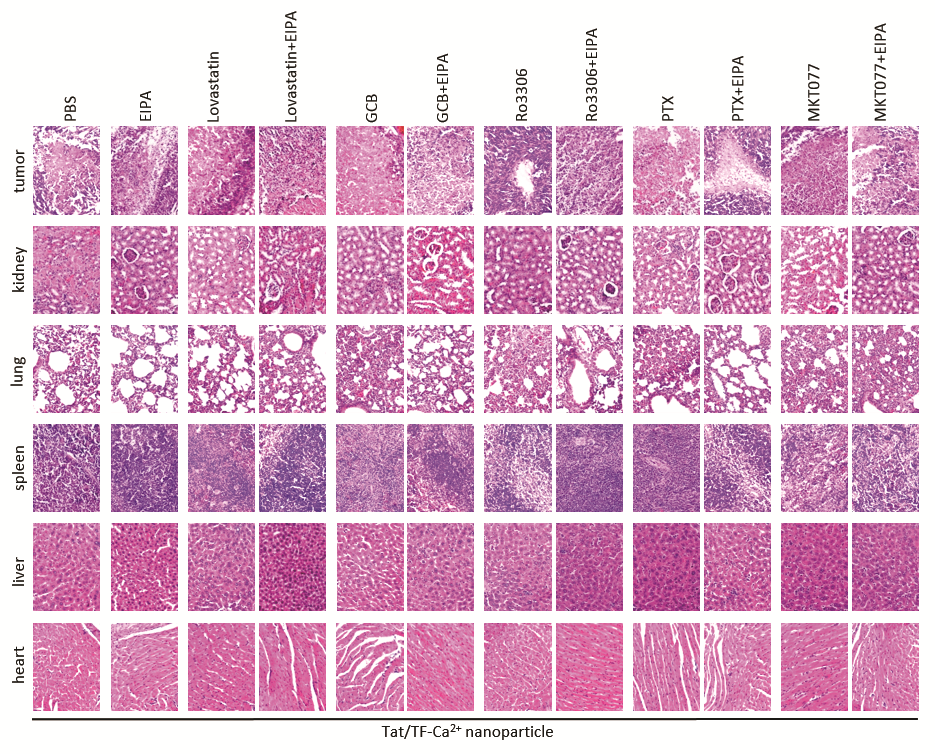


**Fig. S13** **H&E staining of hearts, livers, spleens, lungs, kidneys and tumor tissues from mice with different treatments**.
